# Hierarchical organization of spontaneous co-fluctuations in densely sampled individuals using fMRI

**DOI:** 10.1162/netn_a_00321

**Published:** 2023-10-01

**Authors:** Richard F. Betzel, Sarah A. Cutts, Jacob Tanner, Sarah A. Greenwell, Thomas Varley, Joshua Faskowitz, Olaf Sporns

**Affiliations:** Department of Psychological and Brain Sciences, Indiana University, Bloomington, IN, USA; Program in Neuroscience, Indiana University, Bloomington, IN, USA; Cognitive Science Program, Indiana University, Bloomington, IN, USA; Network Science Institute, Indiana University, Bloomington, IN, USA; School of Informatics, Computing, and Engineering, Indiana University, Bloomington, IN, USA

**Keywords:** Time-varying connectivity, Edge-centric networks, Network states

## Abstract

Edge time series decompose functional connectivity into its framewise contributions. Previous studies have focused on characterizing the properties of high-amplitude frames (time points when the global co-fluctuation amplitude takes on its largest value), including their cluster structure. Less is known about middle- and low-amplitude co-fluctuations (peaks in co-fluctuation time series but of lower amplitude). Here, we directly address those questions, using data from two dense-sampling studies: the MyConnectome project and Midnight Scan Club. We develop a hierarchical clustering algorithm to group peak co-fluctuations of all magnitudes into nested and multiscale clusters based on their pairwise concordance. At a coarse scale, we find evidence of three large clusters that, collectively, engage virtually all canonical brain systems. At finer scales, however, each cluster is dissolved, giving way to increasingly refined patterns of co-fluctuations involving specific sets of brain systems. We also find an increase in global co-fluctuation magnitude with hierarchical scale. Finally, we comment on the amount of data needed to estimate co-fluctuation pattern clusters and implications for brain-behavior studies. Collectively, the findings reported here fill several gaps in current knowledge concerning the heterogeneity and richness of co-fluctuation patterns as estimated with edge time series while providing some practical guidance for future studies.

## Author Summary

Edge time series decompose functional connectivity into its framewise contributions. Previous studies have focused on high-amplitude frames (time points when the global co-fluctuation amplitude takes on its largest value), including their cluster structure. Less is known about middle- and low-amplitude co-fluctuations. Here, we address those questions directly, using data from two dense sampling studies. We group peak co-fluctuations of all magnitudes into hierarchical clusters based on their pairwise concordance. At a coarse scale, we find evidence of three large clusters that engage all brain systems. At finer scales clusters dissolve, yielding refined patterns of co-fluctuations involving specific sets of brain systems. We also find an increase in global co-fluctuation amplitude with hierarchical scale. Finally, we comment on the amount of data needed to estimate co-fluctuation pattern clusters.

## INTRODUCTION

The human brain can be modeled as a network of functionally interconnected brain regions (Bassett & Sporns, 2017). Although functional connectivity (FC) can be estimated using a range of measures—such as spectral coherence, mutual information, and synchronization—in the majority of fMRI analyses, the weights of functional connections are defined as the bivariate correlation between two regions’ activity time series (Reid et al., 2019).

Recent work has demonstrated that a static correlation between two time series, that is, a functional connection, can be “temporally unwrapped” and precisely decomposed into its time-varying contributions (Esfahlani et al., 2020; Faskowitz, Esfahlani, Jo, Sporns, & Betzel, 2020; Sporns, Faskowitz, Teixeira, Cutts, & Betzel, 2021). This procedure generates a co-fluctuation or “edge time series,” whose elements indicate the magnitude and direction of instantaneous coupling between pairs of regions.

Previous analyses of edge time series have focused on high-amplitude co-fluctuations or “events” (but see Sporns et al., 2021, for an exception). Although events occur briefly and infrequently, the pattern of whole-brain co-fluctuations expressed during these periods necessarily contribute more to the time-averaged FC than lower amplitude frames (Esfahlani et al., 2020). Moreover, high-amplitude co-fluctuation patterns can be partitioned into a small number of recurring clusters or “states” (R. F. Betzel, Cutts, Greenwell, Faskowitz, & Sporns, 2022; Esfahlani et al., 2022; Greenwell et al., 2023), can encode information about subjects’ brain-based fingerprints (Jo, Faskowitz, Esfahlani, Sporns, & Betzel, 2021), and can possibly enhance brain-behavior correlations (Esfahlani et al., 2020).

However, interest in high-amplitude events—including work that predates our own (Allan et al., 2015; Cifre, Zarepour, Horovitz, Cannas, & Chialvo, 2020; Liu, Chang, & Duyn, 2013; Petridou, Gaudes, Dryden, Francis, & Gowland, 2013; Tagliazucchi, Balenzuela, Fraiman, & Chialvo, 2012)—has come at the expense of lower amplitude co-fluctuations. In fact, very little is known about their properties. For instance, do they contain subject-specific information? How much does the inclusion of lower amplitude peaks improve predictions of time-averaged FC? That is, given that it is already established that FC can be approximated from a few high-amplitude frames, does prediction accuracy improve when low-amplitude frames are included in the approximation? Do low-amplitude frames exhibit cluster structure? If so, are low- and high-amplitude clusters similar to one another? What does cluster scale—that is, the number and size of clusters—tell us about the time-varying organization of network-level co-fluctuations?

Here, we directly address those questions. Specifically, we analyze densely sampled data from the MyConnectome project (Laumann et al., 2015; Poldrack, 2017) and Midnight Scan Club (MSC; five hours of data for ten subjects; Gordon, Laumann, Gilmore, et al., 2017; Gratton et al., 2018). Whereas previous studies focused on putative “events”—the highest peaks in the global co-fluctuation signal—here we analyze all co-fluctuation peaks, including those of relatively low amplitude, which we aggregate into clusters using a bespoke hierarchical clustering algorithm. We discover that, while high- and low-amplitude co-fluctuation patterns form inter-mixed clusters, lower amplitude patterns tend to be dissimilar from all other patterns and therefore less likely to participate in cohesive clusters. We investigate the hierarchical clusters in greater detail and show that, at a coarse scale, the majority of co-fluctuation patterns could be explained by three broad clusters that get subdivided and refined at deeper hierarchical levels. Whereas the coarse clusters disclose broad, brain-wide co-fluctuation patterns, fine-scale clusters emphasize co-fluctuations involving distinct combinations of functional systems/networks. Finally, we reveal that accurately estimating cluster centroids requires large amounts of data and that, while coarse clusters “lock in” a basic pattern of FC, predictions of FC benefit from the inclusion of fine-scale clusters. This work is the first to investigate the organization of sub-event co-fluctuations, revealing rich structure while setting the stage for future studies.

## RESULTS

Here, we aimed to characterize co-fluctuation patterns estimated using edge time series. Briefly, this procedure entails z-scoring parcel time series, generating edge time series for every pair of parcels, and calculating the root mean square (RMS) of coactivity at each time point. We then partitioned the time series into trough-to-trough event segments, each of which contained exactly one peak frame—local maxima in the RMS time series. For each segment, we extracted the co-fluctuation pattern expressed during its peak for subsequent analysis. Note that our decision to focus only on peaks was based on previous studies in which we demonstrated that, on a per frame basis, high-amplitude frames explain more variance in static FC than lower amplitude frames and that “troughs”—local minima in the RMS signal—correspond to highly variable, low-signal co-fluctuation patterns (R. F. Betzel et al., 2022; Esfahlani et al., 2020). We also opted to analyze single peak frames rather than the average of all frames within a segment. This is because segments varied in duration (number of frames) and we wanted to avoid comparing co-fluctuation patterns estimated using many samples (long segments) with those estimated from few samples (short segments; see Supporting Information Figure S1 for a comparison). After motion censoring, we detected a total of 3,124 peaks. We further discarded peaks whose prominence (height minus the largest of its temporally adjacent troughs) was less than a value of 0.25 or occurred within 10 s of another peak. This procedure resulted in 1,568 co-fluctuation patterns (whose statistical properties are described in Supporting Information Figure S2; we show analogous statistics for data from the Midnight Scan Club in Supporting Information Figure S3). For all subsequent analyses, we pooled the corresponding co-fluctuation patterns from scans.

### Cluster Structure of Full-Spectrum Co-Fluctuation Peaks

Previous studies demonstrated that high-amplitude co-fluctuations could be clustered into a small set of patterns, each of which recurred over time. However, those studies discarded all but the highest-amplitude frames, that is, putative “events,” and used a clustering algorithm that generated communities corresponding to a single organizational scale. Here, we extend those studies by clustering co-fluctuation patterns of varying amplitudes and examining their cluster structure at multiple hierarchical levels.

**Figure 1.**
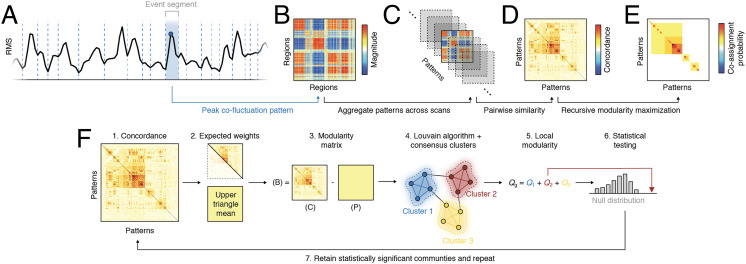
Analysis pipeline. (A) The global amplitude of edge time series (root mean square; RMS) was segmented into motion-free trough-to-trough intervals, or “event segments.” (B) Each segment was represented by the co-fluctuation pattern at its peak. (C) These patterns were aggregated across scans. (D) The similarity between pairs of co-fluctuation patterns was measured with Lin’s concordance. (E) A hierarchical variant of modularity maximization was used to estimate consensus community structure at different scales (resolutions). The multiscale communities were then summarized using a matrix of co-assignment probabilities. (F) Detailed schematic of recursive clustering algorithm. From the full concordance matrix we estimate an expected weight as the mean of these matrices’ upper triangle elements. We calculate a modularity matrix as the observed concordance minus expected weight and submit this to the Louvain algorithm, which we run 1,000 times (different initial conditions) and whose outputs are delivered to a consensus clustering algorithm. We calculate the modularity contribution (local modularity) of each consensus cluster and compare those values with a null distribution generated by randomly permuting consensus community assignments. We retain only those communities whose local modularity is statistically greater than that of the null distribution. The concordance matrices for each such community is returned to step 1 and the algorithm is repeated. This process terminates when the local modularities of all detected consensus communities are consistent with their respective null distribution.

To address this question, we leveraged a hierarchical and recursive extension of the popular community detection method, “modularity maximization” (Blondel, Guillaume, Lambiotte, & Lefebvre, 2008; Newman & Girvan, 2004; see the Materials and Methods section for a detailed description of the algorithm). Our approach is similar to other recursive applications in that we iteratively partition subnetworks until we reach some stopping criterion. Here, we stop when the detected communities have local modularities—for each community, the sum over all within-community edges less their expected weights—that are statistically indistinguishable from a null distribution (Figure 1F). We further excluded small communities (fewer than five elements) and those composed of co-fluctuation patterns from only one scan session. Note that communities correspond to patterns of co-fluctuation that recur across scan sessions. While the individual points assigned to each community may be noisy (they are estimated from single frames), the community/cluster centroids are more stable and spatially smooth.

Here, we apply this algorithm to the similarity matrix estimated from 1,568 peak co-fluctuation patterns. Note that as a measure of similarity we used Lin’s concordance in place of the more common Pearson correlation (Lawrence & Lin, 1989). This concordance measure has been applied previously to brain network data (Shinn et al., 2023) and, in contrast with Pearson’s correlation, which assesses the similarity of two patterns irrespective of their amplitudes, the concordance measure penalizes the similarity score if the amplitudes are mismatched (see the Materials and Methods section for more details).

**Figure 2.**
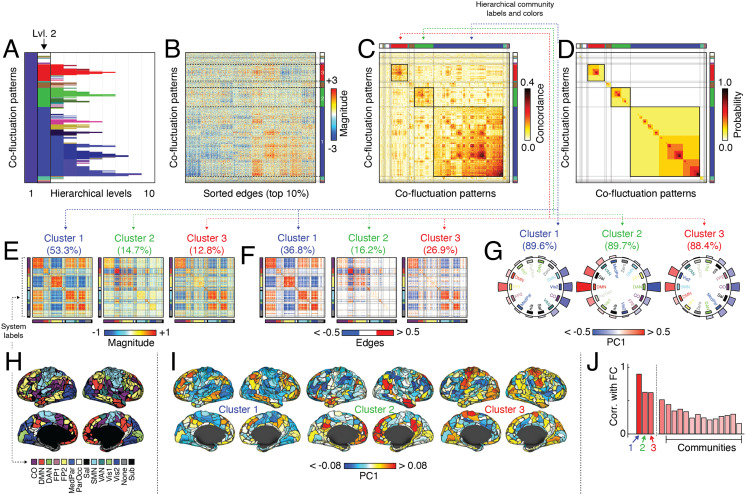
Hierarchical organization of co-fluctuation patterns. Peak co-fluctuation patterns were clustered using a hierarchical analog of modularity maximization. (A) Cluster labels at each hierarchical level. The color associated with each cluster was determined by first assigning each level-3 cluster a unique color (an RGB triplet) using the MATLAB function distinguishable_colors (https://www.mathworks.com/matlabcentral/fileexchange/29702-generate-maximally-perceptually-distinct-colors). For clusters at all other hierarchical levels, we projected their centroids onto the level 3 cluster centroids, rescaling each projection magnitude so that, collectively, they summed to unity. Finally, we assigned each cluster a color as the linear combination of level-3 RGB triplets, each weighted by the corresponding normalized projection magnitude. In this figure, we focus on the coarsest nontrivial hierarchical scale (level 2). This scale is typified by three large clusters, which we color as blue, green, and red. We use this color scheme in all panels. Note that in some cases, the hierarchical communities have similar colors as the system labels obtained from Laumann et al. (2015). We include in-figure text to distinguish system and cluster labels from one another. (B) Top 10% edges, sorted by cluster label. (C) Concordance matrix and (D) co-assignment matrix sorted by community label. (E) Cluster centroids for largest clusters at second hierarchical level. (F) Strongest edges in centroid matrices. (G) We calculated the mean value of PC1 for each brain system, shown here plotted along the perimeter of a circle. In each plot the order of systems differs; those with positive co-fluctuation appear on the left and those with negative, on the right. The text above each plot indicates the variances explained by PC1. (H) MyConnectome system labels from Laumann et al. (2015). (I) Here, we show PC1 for each cluster projected onto the cortical surface. (J) Correlation of mean co-fluctuation patterns with static FC.

We found that the hierarchical clustering algorithm detected 10 distinct hierarchical levels (Figure 2A). At the coarsest scale (hierarchical level 2), we identified three large communities (Figure 2A), each formed by cohesive patterns of co-fluctuations (Figure 2B–D). The largest of these communities (cluster 1) contained 835 patterns (53.3% of all patterns), while the next largest—clusters 2 and 3—contained 231 and 200 patterns (14.7% and 12.8% of all patterns).

Next, we characterized these three clusters in greater detail. For each cluster, we computed its centroid as the mean over all patterns assigned to that cluster (Figure 2E, F). In previous work, we showed that at a timescale of individual frames, co-fluctuation patterns estimated from edge time series can always be viewed as a bipartition of the network into two groups that correspond to collections of nodes whose instantaneous activity levels are above or below their respective means (Sporns et al., 2021). Even at this coarse scale, where clusters represent the average of many individual co-fluctuation patterns, the underlying bipartitions were still apparent. For example, consider cluster 1 (the largest of the three clusters). It is typified by opposed co-fluctuations of cingulo-opercular, visual, attention, and somatomotor networks (group A) with default mode and frontoparietal networks (group B) (Figure 2E). That is, were we to examine regional BOLD data at points in time when this cluster is expressed, we would expect to find the activity of regions in groups A and B to have opposite sign. We can obtain an estimate of this opposed activation pattern by performing a principal component analysis on each of the cluster centroids (Figure 2G). We find that first component (PC1) explains 89.6%, 89.7%, and 88.4% of the variance in each centroid. We can also visualize PC1 by projecting it onto the cortical surface (Figure 2I). Cluster 1, which appears most frequently, also has the strongest correspondence with static (time averaged) FC (Pearson correlation of *r* = 0.90; Figure 2J), suggesting that the prevalence of this activity mode (and corresponding co-fluctuation pattern) helps to lock in the gross connectional features of FC.

Clusters 2 and 3 corresponded to opposed co-fluctuations of default mode with frontoparietal networks (cluster 2) and sensorimotor systems (somatomotor + visual networks) with salience and cingulo-opercular networks (cluster 3). These two communities were also related to static FC, albeit not as strongly correlated (*r* = 0.63 and *r* = 0.62, respectively). Note that the remaining communities, including much smaller communities, were correlated with FC but to a much lesser extent (*r* = 0.30 ± 0.09).

We also performed a series of supplementary analyses. First, to ensure that differences in the correspondence between cluster centroids and static FC were not driven by differences in the amount of data used to estimate each centroid, we repeated this analysis using individual co-fluctuation patterns. In general, the results of this analysis were consistent with those reported here; patterns assigned to Cluster 1 were more strongly correlated with FC compared with those assigned to different clusters (Supporting Information Figure S7). Additionally, we repeated this entire enterprise using MSC data and, despite different acquisition parameters and amounts of data, again observed consistent results (see Supporting Information Figures S8, S9, and S10).

As a final supplementary analysis, we assessed to what extent these results depend on our decision to cluster peaks of the RMS signal as opposed to nearby, but off-peak, frames. To address this question, we resampled the full set of 1,568 peak co-fluctuation patterns by selecting frames from within the same trough-to-trough segment but with a random offset (Supporting Information Figure S4A). Using these off-peak frames, we calculated a null concordance matrix that we compared with the observed matrix. We repeated this process 1,000 times and, for every pair of co-fluctuation patterns, estimated the probability that their null concordance was at least as large as the observed. In general, we found that stronger concordance values corresponded to small *p* values (Spearman’s rank correlation; *ρ* = −0.87, *p* < 10^−15^; Supporting Information Figure S4E). That is, if two peak co-fluctuation patterns were highly concordant, any movement away from their respective peaks resulted in decreased concordance. Because high-concordance pairs tended to be assigned to the same cluster, small *p* values were concentrated within clusters (Supporting Information Figure S4D), suggesting that the detected clusters would be systematically disrupted had we elected to cluster non-peak frames.

Collectively, this analysis of the coarsest level of co-fluctuation patterns generates clusters whose centroids are consistent with those reported in our previous paper (R. F. Betzel et al., 2022). However, unlike that study, the hierarchical clustering algorithm used here allows us to investigate increasingly refined and more exclusive communities. We explore these communities in the next section.

### Subdivisions of Coarse-Scale Community Structure

Our hierarchical clustering approach generates a nested and multiscale description of peak co-fluctuation patterns. In the previous section we focused a single hierarchical level (the coarsest nontrivial partition). Here, we investigate the “children” of those coarse “parent” clusters. For practical reasons, we focus our investigation on subdivisions of clusters 1 and 2 from hierarchical level 2 (described in the previous section). Comparing clusters across hierarchical levels necessitates a naming convention that not only distinguishes a cluster from other clusters in its own hierarchical level, but indicates the level in which it was detected. We now refer to clusters using the convention hierarchical_level.cluster_number. So cluster 1 in hierarchical level 2 would be referred to as “cluster 2.1.” Note that cluster numbers are reused across levels; that is, cluster label 1 will appear in all layers, but will be distinguishable by the prefixes 2.1, 3.1, 4.1, and so on.

**Figure 3.**
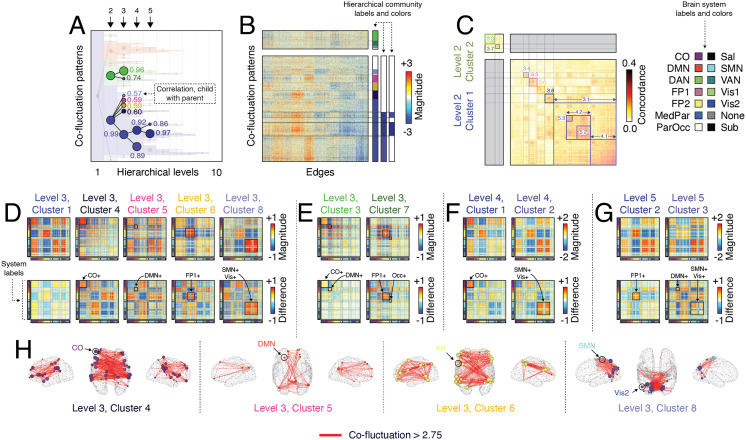
Exploring hierarchical relationships among communities. Previously we investigated a specific hierarchical level of community structure (A). Here, we investigate subdivisions of those communities, focusing on what had previously been termed “cluster 1” and “cluster 2” at the second hierarchical level (we refer to these as clusters 2.1 and 2.2 following the convention hierarchical_level.cluster_number). We overlap a dendrogram over the community label matrix to highlight the divisions of those communities at levels 3, 4, and 5. The text next to each node in the dendrogram denotes the correlation of the corresponding co-fluctuation pattern with its parent. (B) A zoomed-in version of edge co-fluctuation weights for clusters 2.1 and 2.2 at the second hierarchical level, highlighting their subdivisions. (C) Concordance matrix ordered by communities. (D) Mean co-fluctuation patterns (centroids) for subdivisions of cluster 2.1 at level 3. The top row depicts sub-cluster centroid and the bottom row depicts the mean difference of children co-fluctuation patterns with their respective parents. Panels E–G depict analogous matrices for subdivisions of clusters 2.2, 3.1, and 4.1. (H) Edges with the strongest co-fluctuation magnitude (>2.75) for clusters 3.4, 3.5, 3.6, and 3.8.

We find that cluster 2.1, which we described in the previous section, fragments into five distinct communities in the third hierarchical level (Figure 3A–C). The first and largest of these communities, cluster 3.1, represents a refined version of its parent (Figure 3D) in which positive and negative co-fluctuations are reinforced, strengthening their weights. In fact, of the five sub-clusters, this one maintains the strongest similarity to its parent (*r* = 0.99). Incidentally, we observe similar behavior for all three of the large clusters detected in hierarchical level 2. That is, we find evidence of sub-clusters that strongly resemble their parent, but simply increase the magnitude of the strongest positive and negative co-fluctuations (see Supporting Information Figure S11).

The next four clusters (3.4, 3.5, 3.6, and 3.8), however, reflect distinct subcomponents of their mutual parent. Specifically, each cluster exhibits strengthened co-fluctuations within specific functional systems. For instance, cluster 3.4 corresponds to strengthened co-fluctuations among cingulo-opercular regions, while clusters 3.6, 3.7, and 3.8 correspond to increases among default mode, frontoparietal, and the sensorimotor complex (composed of visual and somatomotor systems). Notably, these subdivisions maintain a weaker correspondence with their parent (mean correlation of *r* = 0.59 ± 0.01). We show the top co-fluctuations (edges) for these four clusters in Figure 3H.

Interestingly, cluster 3.1 underwent further refinement in hierarchical levels 4 and 5. Clusters 4.1 and 4.2 reflect increased coupling of cingulo-opercular and a somatosensory complex to themselves, respectively (Figure 3F), while clusters 5.2 and 5.3 split cluster 4.2 into distinct communities that reflect increased coupling of frontoparietal regions to themselves and, separately, the default mode and sensorimotor complex to themselves (Figure 3G).

We also found that cluster 2.2 could be further subdivided. At the coarsest level, this cluster corresponded to opposed co-fluctuations of default mode regions with cingulo-opercular and dorsal attention regions. Of its two sub-clusters, the first (cluster 3.3) could be considered a refinement and continuation of the previous coarser cluster. This cluster also maintained a strong correspondence with its parent (*r* = 0.96). In contrast, cluster 3.7 decoupled the frontoparietal network from the default mode and cingulo-opercular systems (Figure 3F).

**Figure 4.**
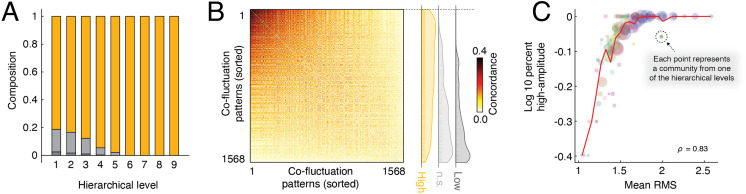
Cluster composition and similarity. At each hierarchical level, we identified the “peak type” of all frames that were not pruned away by the hierarchical clustering algorithm (“high-amplitude,” “not significant” (n.s.), or “low-amplitude”). (A) Composition of all patterns by peak type. Note that as clusters become more exclusive, they tend to be dominated by high-amplitude frames. (B) Concordance matrix ordered by mean similarity of co-fluctuation patterns to one another (greatest to least). The panel to the right displays the relative positions of high-/low-amplitude and not significant patterns. Note that the low-amplitude and not significant patterns are concentrated near the bottom. (C) Assigning patterns a peak type is a discretization of patterns’ RMS values. Here, we show that fraction of frames assigned to a given cluster labeled high-amplitude is tightly correlated with the cluster’s mean RMS. The size of points is proportional to the number of patterns assigned to that community. Each point corresponds to a community detected by the hierarchical clustering algorithm. The size of each point is proportional to the logarithm of the number of nodes assigned to that community and the color comes from the colormap in Figure 2A.

The hierarchical clustering framework also allowed us to explore the composition of clusters at different levels. Specifically, we assessed the extent to which clusters were composed of high-, middle-, or low-amplitude co-fluctuation patterns. We found that, at finer scales, high-amplitude “events” acquired a greater share of the detected clusters (Figure 4A). This observed effect belied a more general and continuous relationship between the similarity of co-fluctuation patterns to one another and their RMS. We found that high-amplitude co-fluctuation patterns tended to have greater levels of similarity to other high-amplitude frames compared with lower-amplitude frames (ANOVA, *F*(2) = 108.8, *p* < 10^−15^; post hoc *t* +tests comparing high-amplitude frames with middle- and low-amplitude frames, *t*(1,527) = 13.2 and *t*(1,311) = 7.1, maximum *p* = 2.3 × 10^−12^; Figure 4B).

Note that the infrequency of low-amplitude peaks is due, in part, to the fact that low-amplitude frames were more likely to be censored because of high levels of in-scanner movement and thresholding based on relative RMS, but also because of a selection bias (we only sampled *peak* co-fluctuations, which are necessary local maxima with RMS greater than most non-peak frames; see Supporting Information Figure S5 and Supporting Information Figure S6 for similarity of peaks to temporally proximal frames).

We note that we also examined clusters from the perspective of network “templates” as described in Sporns et al. (2021; see Supporting Information Figure S12). Briefly, each co-fluctuation pattern forms at most two modules—one composed of nodes whose instantaneous activity is greater than 0 and the other whose activity is less than 0. This suggests that canonical brain systems—such as default mode, visual, and control networks—cannot be collectively expressed at any given instant (only two systems can be present at one time). Rather, brain systems arise from the superposition and averaging of framewise bipartitions. Indeed, the temporal mean of framewise co-assignment matrices—that is, elements are 1 if nodes *i* and *j* are in the same module and 0 otherwise—is almost perfectly aligned static FC and necessarily recapitulates the brain’s canonical system-level architecture. Here, we extend this work by showing that this same effect holds for cluster centroids and not only static FC. Additionally, we match peak activation patterns to a set of predefined “system templates”—bipartitions of canonical systems—and report similar results. See the Materials and Methods section for more details of this analysis.

Collectively, these observations expose the rich, multiscale, and hierarchical organization of peak co-fluctuation patterns. These findings elaborate upon the clusters disclosed in the previous section and earlier papers.

### Module Statistics

One of the primary aims of this manuscript was to investigate clusters of peak co-fluctuations to better understand, specifically, the contribution of low- and middle-amplitude peaks. To address these questions, we analyzed MyConnectome data—a dense sampling study of a single brain (Laumann et al., 2015). One of the advantages of analyzing so much data from a single individual is that we can assess how much data—in terms of time and scan sessions—is necessary to accurately estimate network properties. Previous studies have used these same data to understand how much data is required to estimate static FC. Here, we take an analogous approach so that we can better understand how much data is required to estimate cluster centroids.

To do this, we iteratively split the complete dataset (84 scans) into two random subsets comprising 42 scans each. We then select one scan at random from one of the subsets, and, using only those data, estimate centroids for each of the clusters detected using the full set of data. We then compare those centroids with those estimated using all of the data in the other subset. We then repeat this process after we add in a second scan’s worth of data, then a third, a fourth, and so on, until we have incorporated all of the data available in both subsets. This entire process then gets repeated using a different random bipartition of scans.

**Figure 5.**
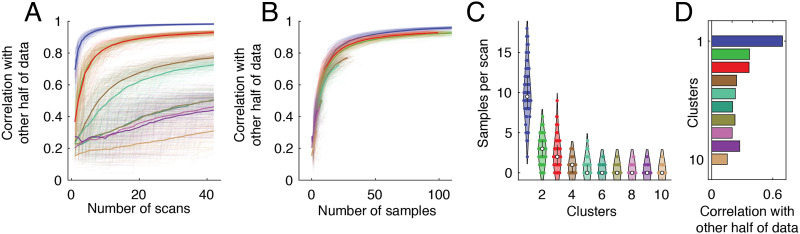
Amount of data required for accurate estimates of cluster centroids. We repeatedly and randomly split the 84 scans into two groups (42 scans each). For group 1 we used all available data to estimate cluster centroids. For group 2, we estimated centroids using data from one random scan and sequentially incorporated data from additional scans. At each step, we calculated the similarity of cluster centroid estimates from group 2 with estimates from group 1. (A) Similarity for clusters 1–10 and hierarchical level 3 as a function of number of scans. (B) Similarity as a function of samples. (C) Number of times that each cluster appears in a given scan. (D) To further control for differences in the number of samples and to assess whether some clusters were composed of patterns that were inherently more similar to their mean, we calculated the mean correlation of individual co-fluctuation patterns in one half of the data with the centroids from the other half.

This procedure allows us to estimate how much data is required to achieve a fixed similarity value. First, we measure the amount of data in units of scans. With the exception of the largest cluster, the similarity curves for the smaller clusters never clearly asymptote (Figure 5A). That is, even after 40 scans, we would expect there to be nontrivial levels of variability in our estimates of cluster centroids.

To understand why this happens, we need to change our unit for measuring the amount of data from “scans” to “samples” and also estimate the baseline frequencies with which each cluster type occurs. In general, we find that co-fluctuation patterns labeled as cluster 1 occur, on average, 9.9 ± 3.4 times per scan (Figure 5C). The next most frequently occurring cluster appears only 2.75 ± 1.6 times per scan. In other words, we expect that each additional scan would yield ≈10 new instances of cluster 1 but between 2 and 3 instances of cluster 2. Therefore, if our aim were to acquire a fixed number of samples of a cluster 2 compared with cluster 1, we would require proportionally four times as many scans. Indeed, when we recreate Figure 5A where units are now in number of samples rather than number of scans, we find that the similarity curves for all clusters overlap (Figure 2B).

Collectively, these results suggest that a key limiting factor in accurately estimating cluster centroids is the relatively low frequencies with which some of the smaller states occur. Our results also suggest that a key factor contributing to variability in connectivity patterns from one day to another might be the frequency with which different cluster patterns appear.

### Linking FC and Hierarchical Depth

**Figure 6.**
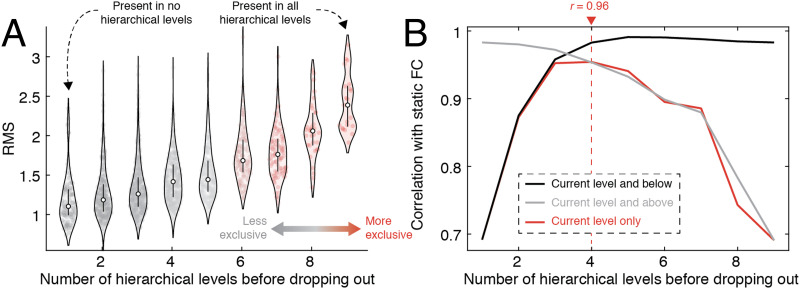
Linking hierarchical levels to FC. We calculated the deepest level of the hierarchy to which each co-fluctuation pattern was assigned (the number of levels in which it appeared before being pruned away). (A) RMS of co-fluctuation patterns, grouped by hierarchical depth. (B) The correlation of mean co-fluctuation patterns with FC. We compared three estimates of the mean: using the current level and below, the current level and above, and the current level only.

The hierarchical procedure yields a progressively sparse perspective on recurring co-fluctuation patterns, with higher levels of the hierarchy appearing more exclusive and containing progressively fewer co-fluctuation patterns but ones that form extremely tight and cohesive clusters (Figure 6A). Because previous studies have linked co-fluctuation patterns to FC, this hierarchical perspective allows us to assess at what hierarchical level (and by extension, what level of exclusivity) do co-fluctuation patterns most closely correspond to FC.

To address this question, we assigned each co-fluctuation pattern a score indicating its “hierarchical depth.” That is, the number of hierarchical levels in which that pattern was present and included in a community. Then we followed the procedure outlined in R. F. Betzel et al. (2022) where, separately for each hierarchical level, we calculated the average co-fluctuation pattern across all patterns assigned to that level, and computed the correlation of that matrix with static FC (based on their upper triangle elements). We also repeated this procedure using a cumulative approach (starting with patterns at the highest level, gradually incorporating patterns from lower levels until all patterns were included) and a reverse cumulative approach (starting with the coarsest scale, gradually peeling away coarser and coarser partitions until only the most exclusive patterns were included).

In general, we found a statistically significant correspondence between FC and co-fluctuation patterns at all hierarchical levels using all three methods for estimating the mean co-fluctuation pattern (minimum *r* = 0.69; *p* < 10^−15^). Using patterns from individual levels only, we found that this correspondence peaked at the fourth hierarchical level (*r* = 0.96), suggesting that the correspondence with FC is maximized when including not only the highest amplitude co-fluctuations, but weaker and less exclusive communities as well. We observed a similar trend using the cumulative and reverse-cumulative approaches—starting with exclusive clusters and including patterns assigned to less exclusive clusters led to improvements in the correspondence, as did starting with all patterns and pruning away weaker patterns. We find similar results with MSC data (see Supporting Information Figure S13).

Collectively, these observations build on results from our previous studies, noting that high-amplitude co-fluctuations are indeed correlated with FC, but that this correlation can be improved upon by including more heterogeneous and slightly weaker co-fluctuation patterns. That is, the lower amplitude patterns, which are more variable, effectively “sculpt” the more stereotypical cofluctuation pattern driven by high-amplitude events, enhancing the diversity of co-fluctuations and improving the correspondence with static FC. Moreover, we find that the correspondence is maximized at an intermediate level, suggesting that different hierarchical scales are differentially informative about FC.

## DISCUSSION

In this paper we build on previous studies of edge time series. Namely, we focused on the statistics of peaks in the RMS time series and the clusters formed by the co-fluctuation patterns expressed during the peaks. We developed a bespoke multiscale clustering algorithm to construct a hierarchy from peak co-fluctuation patterns and investigated clusters at all scales, ranging from coarse clusters that included most patterns to exclusive clusters composed of only a small number of patterns. Finally, we assessed how the amount of data impacts estimates of clusters and their centroids. Collectively, this work addresses several gaps in knowledge and further demonstrates the utility of edge analyses for fMRI data.

### Co-Fluctuation Patterns Are Hierarchically Organized in Time

A large body of work has shown that nervous systems exhibit multiscale, hierarchical organization (R. F. Betzel & Bassett, 2017; Meunier, Lambiotte, Fornito, Ersche, & Bullmore, 2009). Overwhelmingly, this work has focused on hierarchical *spatial* structure, in which neural elements are organized into modules within modules within modules, ad infinitum (Ashourvan, Telesford, Verstynen, Vettel, & Bassett, 2019; Bassett & Bullmore, 2009; Hilgetag & Goulas, 2020; Stam & van Straaten, 2012; Zhou, Zemanová, Zamora, Hilgetag, & Kurths, 2006).

In contrast, there are fewer papers that focus on hierarchies in time. This is not to say that the temporal organization of nervous systems—and functional brain networks, in particular—has gone uncharacterized. In fact, the opposite is true; time-varying connectivity analyses have come to occupy an increasingly large share of contemporary network neuroscience and connectomics (Allen et al., 2014; Bassett et al., 2011; Finc et al., 2020; Leonardi, Shirer, Greicius, & Van De Ville, 2014; Lurie et al., 2020; Sadaghiani, Brookes, & Baillet, 2022; Strindberg, Fransson, Cabral, & Ådén, 2021) (despite several papers that cast doubt on the very premise that network change can be measured with fMRI; Laumann et al., 2017; Liégeois, Laumann, Snyder, Zhou, & Yeo, 2017).

One of the key findings in the time-varying connectivity literature is that brain networks appear to traverse a series of “network states”; that is, a pattern of connectivity approximately persists for some period of time before giving way to a new pattern of connectivity. There also exists mounting evidence that these states are revisited across time within an individual and shared across subjects at the population level (Cornblath et al., 2020; Fiorenzato et al., 2019; Gonzalez-Castillo et al., 2015; Medaglia et al., 2018; Rashid, Damaraju, Pearlson, & Calhoun, 2014).

In most state-based analyses, time-varying estimates of network structure are usually obtained from sliding-window methods. The sliding-window approach, however, represents only one strategy for obtaining (smoothed and temporally imprecise) estimates of time-varying connectivity (Hindriks et al., 2016; Leonardi & Van De Ville, 2015; Zalesky & Breakspear, 2015). Recently, we proposed a method for tracking “instantaneous connectivity” across time, obviating the need for sliding windows (Esfahlani et al., 2020; Faskowitz et al., 2020). Note that we later discovered that this method had been reported at least once before, but had been applied in a narrow context (van Oort et al., 2016). In contrast with the smooth variation observed using sliding windows, edge time series exhibited “bursty” behavior—long periods of quiescence punctuated by brief high-amplitude events. The co-fluctuation patterns coincident with events were strongly correlated with static FC (aligned with earlier findings; Allan et al., 2015; Cifre et al., 2020; Petridou et al., 2013; Tagliazucchi et al., 2012), contained subject-identifying features, and, in an exploratory analysis, strengthened brain-behavior correlations (Esfahlani et al., 2020). Note that in these first studies, we treated edge time series as an instantaneous estimate of time-varying connectivity. However, edge time series can be viewed more generally as a decomposition of any of the correlation between any two variates, irrespective of whether they are, in fact, time series.

Following our initial work, we showed that high-amplitude co-fluctuations could be partitioned into at least two distinct clusters or “states.” These states were shared at the group level, but refined individually, leading to the personalization of subjects’ FC patterns (R. F. Betzel et al., 2022; Greenwell et al., 2023). This two-state description, however, was a direct consequence of the method used to detect states and precluded the possibility that co-fluctuation patterns were organized into clusters at multiple scales or hierarchically. This type of temporal organization, in which broad “meta-states” could be subdivided into a series of smaller states, has been observed in other contexts, such as clusters of independent components (Doucet et al., 2011; Miller et al., 2016; Smith et al., 2012) or states estimated using hidden Markov models (Vidaurre, Smith, & Woolrich, 2017). However, it was unclear whether edge time series exhibited analogous temporal structure.

To address this question, we investigated data from the MyConnectome project. Our rationale for selecting this dataset was that, with more than 10 hr of resting-state data, the MyConnectome project gives us the best chance to detect infrequent states, if they exist, and to obtain better estimates of the states that occur more frequently. To investigate co-fluctuation states, we developed a hierarchical clustering algorithm built upon recursive application of the familiar modularity maximization algorithm (Newman & Girvan, 2004), allowing us to obtain estimates of large “meta-states” but also smaller, more refined states.

We found evidence of three large clusters of co-fluctuation patterns that persisted over multiple hierarchical levels, gradually refining their organization. Notably, the centroids of these clusters were consistent with those reported in our previous work (R. F. Betzel et al., 2022), and they were aligned with other recent findings. For instance, our clusters delineate task-positive and -negative systems (Fox et al., 2005; Golland, Golland, Bentin, & Malach, 2008), recapitulate spatial modes of variation in resting-state data time series (Bolt et al., 2022), and closely resemble components of so-called functional gradients (Margulies et al., 2016), which are frequently interpreted in terms of cognitive hierarchies (Mesulam, 1998).

Importantly, we also found that these large clusters could be meaningfully subdivided into smaller, increasingly nuanced patterns of co-fluctuation. This observation has important implications for how we interpret static FC, but also for our understanding of brain dynamics and interareal communication. Because edge time series are an exact decomposition of FC into framewise contributions, the average across peak co-fluctuations serves as an approximation of FC. While this pattern-level estimate is generally very accurate, it offers no compression (each individual pattern is needed). By clustering patterns, we reduce the description length of co-fluctuation patterns while, hopefully, still generating a good approximation of FC. Indeed, we find that this is the case, with different hierarchical levels and clusters offering variable predictions. Of particular interest is the observation that mid-hierarchy co-fluctuations actually outperform other levels. This is because clusters of co-fluctuation patterns at coarse scales are too general to recapitulate details of FC connectivity patterns, while clusters at the finest scales are too specific. Note that the aim of comparing cluster centroids to static FC was to understand how individual clusters uniquely contribute to the time-averaged FC pattern. The aim is not to maximize the distance between cluster centroids and static FC. Indeed, given the observations made here and elsewhere (R. F. Betzel et al., 2022; Esfahlani et al., 2020), it appears difficult for recurring co-fluctuation patterns to be wholly unrelated to static FC. This observation is, perhaps, not so profound, as edge time series are mathematically precise decompositions of FC and, if one were to average subsets of time points, the mean co-fluctuation pattern should approximate static FC.

FC is frequently interpreted as evidence of communication or coordination between pairs of brain regions (Avena-Koenigsberger, Misic, & Sporns, 2018; Reid et al., 2019). This interpretation is evident when we consider the brain’s static system organization; that is, its division into subnetworks like the default mode, visual, and attentional systems. We generally think of these cohesive modules as reflecting the outcome of a segregated and functionally specialized process. In previous studies, however, we demonstrated that, as measured with fMRI, no more than two modules can be “engaged” at any single point in time, implying that the brain’s static system-level architecture is a consequence of dynamically fluctuating bipartitions that, occasionally, do not resemble any of the frequently discussed brain systems (Sporns et al., 2021). Moreover, if we think of static FC as a reflection of interareal communication, then each bipartition—and especially those that occur during peaks, when the co-fluctuation magnitude is much stronger than nearby frames—may reflect a communication event. Our findings, here, suggest that these instants of communication are highly structured in space and time. Spatially, we identify a richer repertoire of co-fluctuation patterns than had previously been reported (more clusters) and show that, while these patterns can involve the entire cerebral cortex, they also can engage specific subsets of systems. Our findings also suggest that these patterns occur intermittently but recur across time. Thus, the brain’s temporal trajectory as defined by edge time series is low-dimensional, but also bursty.

### Hierarchies Contain Heterogeneous Co-Fluctuation Patterns of Similar Amplitude

In most previous analyses of edge time series, emphasis was placed on high-amplitude frames (R. F. Betzel et al., 2022; Esfahlani et al., 2020; Greenwell et al., 2023). That is, instants in time where the global co-fluctuation amplitude was disproportionately large. The rationale for doing so was that, because FC is literally the mean of an edge time series, frames with large amplitude *must* contribute more to the average, and frames where many edges have large amplitude necessarily contribute more to the overall FC pattern. However, these high-amplitude periods are rare, and while on a per frame basis they contribute more than middle and lower amplitude frames, they number far fewer. Moreover, they represent only the tail of a distribution and ignore low-amplitude frames, which tend to be more susceptible to motion artifacts (R. F. Betzel et al., 2022), but also middle-amplitude frames, about which less is known.

Here, we find that the overall magnitude of co-fluctuations scales with hierarchical level. That is, the co-fluctuation patterns that make up the most exclusive and highest level of the hierarchy tend to be composed of those with the greatest overall amplitude, while lower amplitude patterns populate the intermediate levels of the hierarchy. This observation is analogous to recent findings, reporting a graded link to FC (Ladwig et al., 2022). However, our findings also suggest that nuance is necessary in describing links between amplitude and FC and that, at every hierarchical level, there exists structured heterogeneity of co-fluctuation patterns, that is, they can be grouped into clusters.

### Accurate Estimates of Cluster Centroids Require Lots of Data

One of our key observations is that, if we want to accurately estimate cluster centroids, we require large amounts of data. For some of the smaller and less frequently appearing clusters, this amount is prohibitively large and infeasible for most fMRI studies (greater than 7 hr). This observation is in line with other studies showing that a major source in the variability of functional brain networks is the amount of data (Gratton et al., 2018; Laumann et al., 2015; Noble et al., 2017). In fact this effect gets amplified when estimating co-fluctuations; while a typical scan session samples brain activity at hundreds of time points, a much smaller fraction of those will correspond to peaks.

While this effect can be viewed as a limitation, it also serves as a potential explanation for observed variability in network architecture from one day to the next. Because FC is the average of co-fluctuation patterns across time, differences in cluster frequencies across scan sessions will, necessarily, correspond to differences in FC weights.

### Limitations and Future Directions

One of the limitations of this study is its reliance on “dense-sampling” datasets. The rationale for studying these types of data (rather than cross-sectional datasets) comes from our previous studies (R. F. Betzel et al., 2022), where we demonstrated that recurring co-fluctuation patterns, while similar across individuals, are also individualized. Accordingly, we aimed to study co-fluctuation patterns at the individual level rather than at the cohort level, where cluster centroids, because they are composed of patterns from many individuals, may not be representative of any of those individuals. However, while dense-sampling studies allow researchers to characterize individuals in great detail, they make it challenging to generalize to the population/cohort level. Nonetheless, there is value in examining effects at that level, as many populations are not amenable to dense-sampling designs, necessitating cross-sectional analysis. Future studies should extend this work to larger cross-sectional datasets, such as the Human Connectome Project.

Recent papers have shown that some of the apparently “dynamic” features of edge time series, including the emergence of events, can be explained parsimoniously by properties of the static FC matrix, such as its eigenspectrum (Matsui, Pham, Jimura, & Chikazoe, 2022; Novelli & Razi, 2022). First, we note, that this does not change the view of edge time series as a decomposition of FC; the mean of an edge time series is still exactly that edge’s weight. Second, even if one were to accept that the peak co-fluctuations do not occur “dynamically” but reflect sampling variability around a stationary correlation structure, we can still view edge time series (and clusters of co-fluctuation patterns), from an explanatory perspective, analogous to how we interpret the results of a principal component analysis (where components correspond to modes of variability that explain linear dependencies in the larger dataset). While there is an indisputable mathematical equivalence between fluctuations in edge time series and static FC, there remain dynamic features that are not easily dismissed. For instance, it was observed that edge time series synchronize across individuals during movie-watching (Esfahlani et al., 2020); this effect would be unanticipated if edge time series were stochastic fluctuations around a stationary correlation structure.

There exist other overarching philosophical disputes concerning the origins of and appropriate null models for edge time series (and task-free brain activity more generally). For instance, observed fMRI BOLD time series are generated by an underlying dynamical system constrained by anatomical connectivity (Cabral, Kringelbach, & Deco, 2017; Ritter, Schirner, McIntosh, & Jirsa, 2013; Sanz-Leon, Knock, Spiegler, & Jirsa, 2015). That is, there exists an evolution operator that maps a pattern of activity at time *t* to a new pattern at time *t* + 1, and this operator is parameterized by structural connectivity (among other parameters). The activity time series generated by this dynamical system can, of course, be summarized by its correlation structure, that is, its FC. However, FC itself plays no role in determining the evolution of brain activity in the model. That is, FC is a summary statistic, ephiphenomenal, and over short timescales plays no role in shaping the character of ongoing brain activity. Rather, brain activity is shaped by dynamics that are constrained by anatomy. However, many “null” models stochastically generate synthetic fMRI BOLD data given a fixed correlation structure, often estimated from the data themselves (Liégeois, Yeo, & Van De Ville, 2021), circularly presupposing that the observed correlation structure is the driver of itself. In short, while the results reported here do not directly speak to the dynamics of co-fluctuation time series, they set the stage for future studies to perform detailed explorations using generative models grounded in anatomical connectivity (Pope, Fukushima, Betzel, & Sporns, 2021).

As part of this paper, we create or use several tools that might be useful for future studies. First, we used an existing measure of concordance (Lawrence & Lin, 1989) for assessing the similarity of co-fluctuation patterns to one another rather than correlation measures, which are far more common. Our rationale for choosing concordance is that it is sensitive to differences in amplitude. Imagine having two co-fluctuation (or connectivity) patterns; they are identical patterns, but in one case all edge weights are scaled by a number very close to 0 so that, effectively, each weight is 0, but there remains a faint impression of the original co-fluctuation pattern. The correlation of these two patterns is exactly 1, despite the vast difference in amplitude. Their concordance, on the other hand, would be near 0. In short, concordance is a more conservative measure of similarity and could be applied in other contexts to assess the correspondence between connectivity or co-fluctuation matrices.

The second innovation is the multiscale and hierarchical clustering algorithm. It addresses several limitations of community detection methods frequently applied to neuroimaging data. First, unlike single-scale community detection algorithms, it generates multiscale estimates of communities at different resolutions. Note that there are many algorithms and approaches for generating multiscale estimates of communities including varying resolution parameters (Sperry, Kartha, Granquist, & Winkelstein, 2018) or sparsity levels (Gordon et al., 2020), although these approaches do not explicitly establish hierarchical relationships between scales, which our method does. Additionally, and importantly, our approach incorporates an internal null model that makes it possible to reject communities, an important consideration given that descriptive community detection methods can spuriously detect communities without proper statistical controls (Guimera, Sales-Pardo, & Amaral, 2004). Here, we test the local modularity contributions of each community, retaining those where the contribution is significantly greater than that of a chance model. We note, however, that other criteria could be substituted and used to determine whether a community is propagated to the next level. Finally, the algorithm is computationally efficient in comparison to other similar methods (Jeub, Sporns, & Fortunato, 2018). Future analyses should focus on benchmarking this method.

## MATERIALS AND METHODS

### Midnight Scan Club

The description of the Midnight Scan Club dataset acquisition, preprocessing, and network modeling is described in detail in Gordon, Laumann, Gilmore, et al. (2017). Here, we provide a high-level overview. Data were collected from 10 healthy, right-handed, young adult participants (five females; age: 24–34). Participants were recruited from the Washington University community. Informed consent was obtained from all participants. The study was approved by the Washington University School of Medicine Human Studies Committee and Institutional Review Board. This dataset was previously reported in Gordon, Laumann, Gilmore, et al. (2017) and Gratton et al. (2018) and is publicly available at https://openneuro.org/datasets/ds000224/versions/00002. Imaging for each participant was performed on a Siemens TRIO 3T MRI scanner over the course of 12 sessions conducted on separate days, each beginning at midnight. In total, four T1-weighted images, four T2-weighted images, and 5 hr of resting-state BOLD fMRI were collected from each participant. For further details regarding data acquisition parameters, see Gordon, Laumann, Gilmore, et al. (2017).

High-resolution structural MRI data were averaged together, and the average T1 images were used to generate hand-edited cortical surfaces using FreeSurfer (Dale, Fischl, & Sereno, 1999). The resulting surfaces were registered into fs_LR_32k surface space as described in Glasser et al. (2013). Separately, an average native T1-to-Talairach (Talairach, 1988) volumetric atlas transform was calculated. That transform was applied to the fs_LR_32k surfaces to put them into Talairach volumetric space.

Volumetric fMRI preprocessing included slice-timing correction, frame-to-frame alignment to correct for motion, intensity normalization to mode 1000, registration to the T2 image (which was registered to the high-resolution T1 anatomical image, which in turn had been previously registered to the template space), and distortion correction (Gordon, Laumann, Gilmore, et al., 2017). Registration, atlas transformation, resampling to 3 mm isotropic resolution, and distortion correction were all combined and applied in a single transformation step (Smith et al., 2004). Subsequent steps were all completed on the atlas transformed and resampled data.

Several connectivity-specific steps were included (see Power et al., 2014): (a) demeaning and de-trending of the data, (b) nuisance regression of signals from white matter, cerebrospinal fluid, and the global signal, (c) removal of high-motion frames (with framewise displacement (FD) > 0.2 mm; see Gordon, Laumann, Gilmore, et al., 2017) and their interpolation using power-spectral matched data, and (d) bandpass filtering (0.009 Hz to 0.08 Hz). Functional data were sampled to the cortical surface and smoothed (Gaussian kernel, *σ* = 2.55 mm) with 2-D geodesic smoothing.

The following steps were also undertaken to reduce contributions from non-neuronal sources (Ciric et al., 2017; Power et al., 2014). First, motion-contaminated frames were flagged. Two participants (MSC03 and MSC10) had high-frequency artifacts in the motion estimates calculated in the phase encode (anterior-posterior) direction. Motion estimate time courses were filtered in this direction to retain effects occurring below 0.1 Hz. Motion-contaminated volumes were then identified by frame-by-frame displacement (FD, described in Power, Barnes, Snyder, Schlaggar, & Petersen, 2012), calculated as the sum of absolute values of the differentials of the three translational motion parameters (including one filtered parameter) and three rotational motion parameters. Frames with FD greater than 0.2 mm were flagged as motion-contaminated. Across all participants, these masks censored 28% ± 18% (range: 6%–67%) of the data; on average, participants retained 5,929 ± 1,508 volumes (range: 2,733–7,667). Note that in this paradigm, even the worst participant retained almost two hours of data. Nonetheless, we excluded two subjects from all analyses, both of whom had fewer than 50% usable frames in at least five scan sessions (MSC08 in 7/10 and MSC9 in 5/10).

Time courses were extracted from individualized parcellations (see Gordon, Laumann, Adeyemo, & Petersen, 2017, for details). The time series were used for FC estimation and edge time series generation.

### MyConnectome Dataset

All data and cortical surface files are freely available and were obtained from the *MyConnectome Project*’s data-sharing webpage (https://myconnectome.org/wp/data-sharing/). Specifically, we studied preprocessed parcel fMRI time series for scan sessions 14–104. Details of the preprocessing procedure have been described elsewhere (Laumann et al., 2015; Poldrack et al., 2015). Each session consisted of 518 time points during which the average fMRI BOLD signal was measured for *N* = 630 parcels or regions of interest. With a TR of 1.16 s, the analyzed segment of each session was approximately 10 min long.

### Functional Connectivity

Functional connectivity (FC) measures the statistical dependence between the activity of distinct neural elements. In the modeling of macroscale brain networks with fMRI data, this usually means computing the Pearson correlation of brain regions’ activity time series. To calculate FC for regions *i* and *j*, then, we first standardize their time series and represent them as z-scores. We denote the z-scored time series of region *i* as **z***_i_* = [*z*_*i*_ (1), …, *z*_*i*_ (*T*)], where *T* is the number of samples. The Pearson correlation is then calculated as the following:

$$r_{ij}=\frac{1}{T-1}\sum_{t=1}^{T}z_{i}\left(t\right)\cdot z_{j}\left(t\right).$$ (1)

In other words, the correlation is equal to the temporal average of two regions’ co-fluctuation.

### Edge Time Series

We analyzed edge time series data. Edge time series can be viewed as a temporal decomposition of a correlation (functional connection) into its framewise contributions. Note that Pearson correlation is calculated as *r*_*x*,*y*_ = $\frac{1}{T-1}$ ∑_*t*_ *z*_*x*_(*t*) · *z*_*y*_(*t*), where *T* is the number of samples and *z*_*x*_(*t*) = $\frac{x-\mu_{x}}{\sigma_{x}}$ is the z-scored transformation of the time series *x* = [*x*(1), …, *x*(*T*)]. If we omit the summation in our calculation of *r*_*x*,*y*_, we obtain a time series *r*_*x*,*y*_(*t*) = *z*_*x*_(*t*) · *z*_*y*_(*t*), whose elements index the instantaneous co-fluctuation between variates *x* and *y*. Here, we estimated the edge time series for all pairs of brain regions {*i*, *j*}.

### Time Series Segmentation and Peak Detection

In estimating edge time series, we censored all frames with high levels of motion. In addition, we further censored frames that were within two TRs of a high-motion frame. Finally, of the remaining frames, we discarded any temporally contiguous sequences of low-motion frames that were shorter than five TRs. For a given scan session, this procedure induced discontinuous sequences of low-motion data. Aside from z-scoring parcel time series (for which the mean and standard deviation were estimated using all low-motion frames), all subsequent analyses were carried out separately for each sequence.

Let *r*_*ij*_(*t*) be the co-fluctuation magnitude between regions *i* and *j* at time *t*. The magnitude of co-fluctuation at any instance can be calculated as *R*(*t*) = $\sqrt{\sum_{ij\in E}r_{ij}{\left(t\right)}^{2}}$. Here, *E* = {{1, 2}, …, {*N* − 1, *N*}} is the set of all node pairs (edges) and *N* is the total number of nodes (630 for the MyConnectome dataset). We used MATLAB’s findpeaks function to identify local minima in the RMS time series, resulting in 3,124 low-motion, trough-to-trough segments. Within each segment there exists a single peak frame; we then calculated its relative RMS as its height minus the height largest of its neighboring troughs. We retained only those peaks whose relative RMS was greater than 0.25, reducing the number of segments to 1,717. As a final exclusionary criterion, we identified peaks that occurred within 10 s of one another and retained only the peak with the greater relate RMS, further reducing the number of segments to 1,568 (50.1% of the original).

### Lin’s Concordance

In order to detect clusters among peak co-fluctuation patterns, we needed a distance metric to assess their pairwise similarity. A common candidate in human neuroimaging and network neuroscience studies is the Pearson correlation (correlation similarity). However, this measure rescales patterns before computing their similarity (z-score). That is, two co-fluctuation patterns with very different magnitude would be considered highly similar using the correlation metric. Here, however, we explicitly aimed to compare co-fluctuation patterns of differing amplitude and needed a distance/similarity metric sensitive to these differences.

Accordingly, we opted to use Lin’s concordance as a measure of similarity (Lawrence & Lin, 1989). Briefly, this measure simultaneously assesses the similarity between two vectors based on their overall pattern (like the correlation matrix), but also allows vectors to be distinguished from one another if their magnitudes differed. Briefly, the concordance between two vectors, *x* and *y*, is calculated as the following:

$$C_{xy}=\frac{2\cdot Cov\left(x,y\right)}{Var\left(x\right)+Var\left(y\right)+{\left(\mu_{x}-\mu_{y}\right)}^{2}}.$$ (2)

Intuitively, if the two vectors have identical means and variances, then their concordance is equal to their correlation coefficient *r*_*xy*_, which serves as an upper bound. However, if the variances or means of *x* and *y* differ, then *C*_*xy*_ < *r*_*xy*_. Here, we calculate the pairwise concordance between all peak co-fluctuation patterns. This matrix is calculated separately for each subject.

### Recursive Modularity Maximization, Modularity Contributions, and Statistical Tests

We used a community detection algorithm to partition co-fluctuation patterns into hierarchically related clusters. Specifically, we recursively applied modularity maximization to the concordance matrix. The modularity heuristic defines communities as groups of nodes whose density of connections to one another maximally exceeds what would be expected by chance.

In general, the modularity, *Q*, of a partition can be expressed as the sum of contributions made by each community, *c* ∈ {1, …, *K*}, such that

$$Q=\sum_{c=1}^{K}q_{c},$$ (3)

where *q*_*c*_ = ∑_*i*∈*c*,*j*∈*c*_ [*C*_*ij*_ − *P*_*ij*_]. In this expression, *i* and *j* correspond to distinct elements in the network (in our case, peak co-fluctuation patterns). The values of *C*_*ij*_ and *P*_*ij*_ correspond to the observed and expected concordance between those pairs of patterns.

Our algorithm is simple and built upon the modularity maximization framework. For a given concordance matrix, we uniformly set the expected weight of connections equal to the mean of its upper triangle elements (the concordance measure is symmetric). That is, for all {*i*, *j* > *i*} pairs, the expected weight is a uniform constant and equal to *P* = $\frac{2}{N_{p}\left(N_{p}-1\right)}$ ∑_*i*,*j*>*i*_ *C*_*ij*_, where *N*_*p*_ is the total number of peak co-fluctuation patterns detected. The contribution to the total modularity by community *c* can therefore be written as *q*_*c*_ = ∑_*i*∈*c*,*j*∈*c*_ [*C*_*ij*_ − *P*].

Next, we used a generalization of the Louvain algorithm (Blondel et al., 2008) to optimize the modularity *Q*, repeating the procedure 1,000 times with random restarts before obtaining a consensus partition (R. F. Betzel et al., 2017). In general, the consensus partition will contain *K* communities, each of which is associated with a modularity contribution, *q*_*c*_. We compare the observed contribution value against a null distribution generated by preserving the consensus community labels but randomly assigning peak co-fluctuation patterns to communities (10,000 repetitions). We then calculate a *p* value as the fraction of times that the null value was greater than that of the observed value. Communities were propagated to the next level if *p* < 0.05.

If a community survived this statistical test, it was propagated to the next level, where the entire procedure is repeated. This algorithm continues until no detected communities pass the statistical test. The end result is a series of nested communities that can be linked to one another via a dendrogram.

Note that the dendrogram generated by this algorithm creates unambiguous mappings between parent and child clusters. This happens because of the recursive application of modularity maximization to concordance matrices. At each level, concordance matrices are partitioned independently so that elements in their respective sub-clusters overlap with elements in the parent cluster, exclusively.

Note also that we compared the performance of the hierarchical clustering algorithm on observed peak co-fluctuation patterns against peak co-fluctuation patterns obtained under a null model. Specifically, we generate null co-fluctuation patterns by circularly shifting parcel time series independently. This procedure preserves the moments of each time series but destroys the correlation between pairs of time series. We applied this procedure to data from a single MyConnectome project scan session. When applied to the observed data, the hierarchical clustering algorithm detected six hierarchical levels (Supporting Information Figure S14A–D). However, for the circularly shifted data, every single run returned exactly two hierarchical levels, each of which were trivial (the first level was a single community composed of all communities; the second level was entirely empty and contained no statistically significant communities; Supporting Information Figure S14E–G). These observations suggest that when applied to simulated data with no correlation structure (and therefore no clear cluster structure), the hierarchical clustering algorithm is effective in that it falsely detects communities at a very low rate.

### Centroid Analysis

Throughout this report we found it useful to examine individual communities in more detail. One way to summarize a community is by computing the mean co-fluctuation pattern across all patterns assigned to that community, that is, the community’s centroid. The elements of this pattern can be represented as a [*node* × *node*] co-fluctuation matrix.

To better understand the modes of activity that underpin each co-fluctuation pattern, we performed an eigendecomposition of each co-fluctuation matrix, which yielded a series of [*node* × 1] eigenvectors, each associated with an eigenvalue that was linearly proportional to the amount of variance explained by its corresponding eigenvector. We focus only on the eigenvector corresponding to the largest eigenvalue. For the sake of visualization, we sometimes present the largest eigenvector at the system level. This procedure involves identifying the indices of the nodes assigned to each brain system, extracting their values from the [*node* × 1] leading eigenvector and computing the average of these values (see Figure 2G for an example). Note that the procedure does not require that we recalculate eigenvectors/principal components.

### Bipartition Analysis

In a previous study we showed that the co-fluctuation pattern expressed at each moment in time could be partitioned into exactly two communities (a bipartition) based on whether each node’s activity was above or below its mean value (Sporns et al., 2021). These bipartitions effectively retain only the signs of a node’s activity and necessarily discard details about its amplitude. Despite this loss of information, the mean co-assignment of nodes to the same community closely recapitulate the brain’s static connectivity structure We refer to this co-assignment matrix as the “bipartition co-assignment matrix.”

The bipartitions framework also facilitates a straightforward comparison with canonical brain systems. Specifically, brain systems, such as default mode, visual, and control networks, can be represented as bipartitions. The nodes assigned to that system are given a value of 1, while the others are assigned 0. One could also consider combinations of systems, such as by assigning nodes in systems A + B + C a value of 1. The empirical and system bipartitions can be compared with one another directly using the measure of normalized mutual information—values close to 1 indicate a correspondence between the two bipartitions; values near 0 indicate no relationships.

Here, we used the bipartition analysis to relate each peak co-fluctuation pattern with one of 8,191 template patterns (all possible partitions of the 14 systems into two clusters). Each template is, itself, a bipartition and can be represented as a template co-assignment matrix. We then calculate a sum across all template co-assignment matrices where each matrix is weighted based on the relative frequency with which peak co-fluctuation patterns were maximally similar to it. We refer to the resulting composite matrix as the “weighted template.” We also report a related version of this matrix in which we z-score the off-diagonal elements of the weighted templates matrix; we refer to this as the “weighted and z-scored templates” matrix.

## SUPPORTING INFORMATION

Supporting information for this article is available at https://doi.org/10.1162/netn_a_00321.

## AUTHOR CONTRIBUTIONS

Richard Betzel: Conceptualization; Data curation; Formal analysis; Funding acquisition; Investigation; Methodology; Project administration; Resources; Software; Supervision; Validation; Visualization; Writing – original draft; Writing – review & editing. Sarah Cutts: Writing – original draft; Writing – review & editing. Jacob Tanner: Writing – original draft; Writing – review & editing. Sarah Greenwell: Writing – original draft; Writing – review & editing. Thomas Varley: Writing – original draft; Writing – review & editing. Joshua Faskowitz: Data curation; Writing – original draft; Writing – review & editing. Olaf Sporns: Conceptualization; Supervision; Writing – original draft; Writing – review & editing.

## FUNDING INFORMATION

Richard Betzel, National Science Foundation (https://dx.doi.org/10.13039/100000001), Award ID: 076059-00003C. Richard Betzel, National Science Foundation, Award ID: 2023985. Olaf Sporns, National Science Foundation, Award ID: 2023985.

## TECHNICAL TERMS

Functional connectivity: Matrix of all interregional correlations, usually estimated at rest.
Edge time series: A framewise decomposition of a correlation and an estimate of instantaneous co-fluctuations between pairs of neural elements.
Events: Time points when the root mean squared amplitude of edge time series exceeds what is expected under a null model.
Co-fluctuation matrix or co-fluctuation pattern: The set of all interregional edge time series at a given time point; typically represented as a Node × Node matrix.
