# Supplementary material for: Hierarchical organization of spontaneous co-fluctuations in densely sampled individuals using fMRI
Source: Netw Neurosci. 2023 Oct 1;7(3):926–49. doi: 10.1162/netn_a_00321 (PMC10473297; doi:10.1162/netn_a_00321)
Supplement: Supplementary file 1 [file netn-7-3-926-s001.pdf]

Supplement to: Hierarchical organization of spontaneous co-fluctuations in densely-sampled individuals using fMRI

Richard F. Betzel[1−4], Sarah A. Cutts[1,2], Jacob Tanner[3,5], Sarah A. Greenwell[1], Thomas Varley[1,5], Joshua Faskowitz[1], and Olaf Sporns[1−4*]
[1]*Department of Psychological and Brain Sciences,*
[2]*Program in Neuroscience,* [3]*Cognitive Science Program,*
[4]*Network Science Institute,* [5]*School of Informatics, Computing, and Engineering, Indiana University, Bloomington, IN 47405*
(Dated: April 29, 2023)

This document contains supplementary figures to the article entitled "Hierarchical organization of spontaneous co-fluctuations in densely-sampled individuals using fMRI".

* rbetzel @ indiana.edu

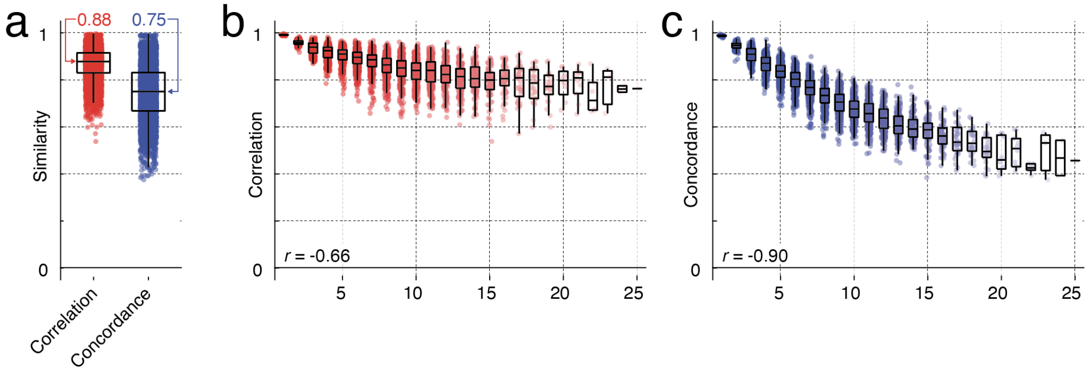

FIG. S1. **Comparing methods for estimating representative co-fluctuation patterns.** In the main text we partitioned time series into trough-to-trough segments and, for each segment, extracted the co-fluctuation pattern with the peak RMS. We considered these patterns as representative of the entire segment. An alternative strategy for estimating representative patterns is to obtain the average co-fluctuation pattern across all frames within a given segment. Here, we compare representative patterns estimated using these two approaches, calculating their similarity to one another using correlation and concordance metrics. (*a*) Similarity of peak and average co-fluctuation patterns. Each point corresponds to a single segment. Panels *b* and *c* show the same data as panel a, but with similarity values ordered by segment duration (number of frames).

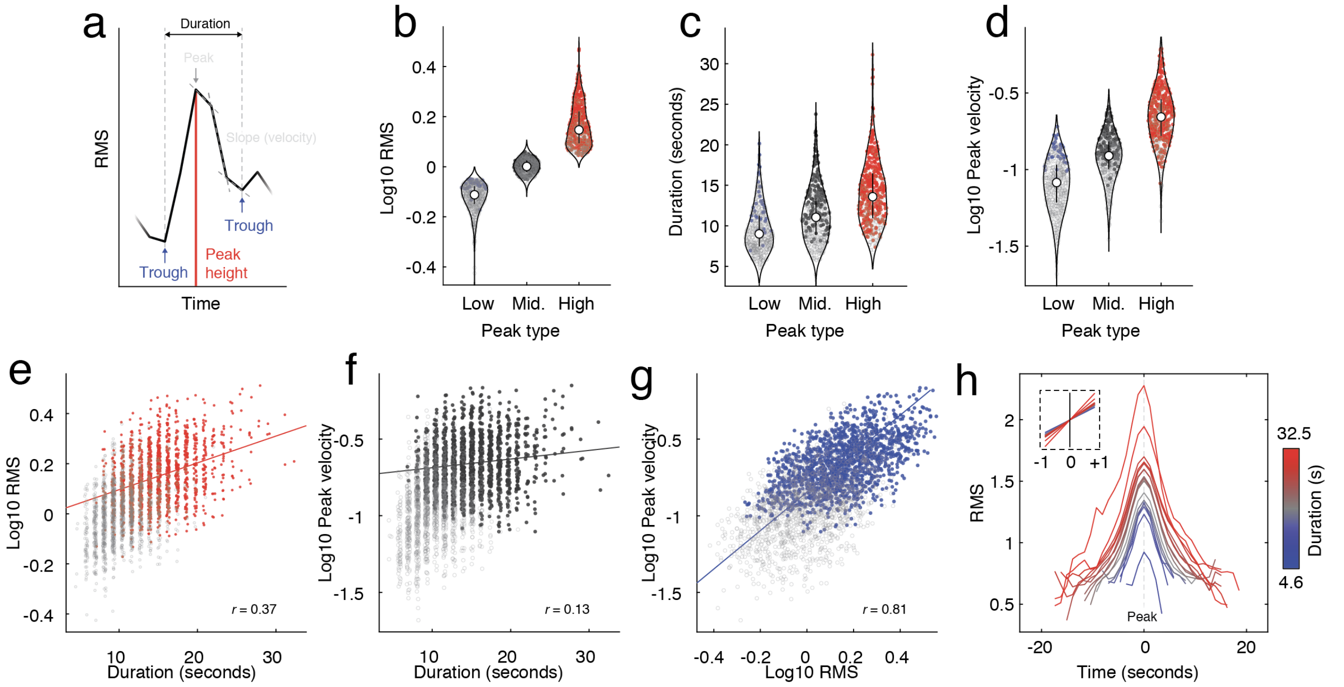

FIG. S2. **Characterizing peak co-fluctuations.** For every peak, we calculated its amplitude (RMS) and duration. Using a procedure developed in our previous work, also classified each peak as a high-, middle-, or low-amplitude frame. In the main text and in all panels we report statistics based on a set of 1568 co-fluctuation patterns that survived a series of quality assessments to reduce the likelihood that they are related to motion or reflect background stochastic fluctuation. In all plots, these 1568 points are opaque. For completeness, we also include the remaining 1556 detected peaks that were discarded. These points are depicted as gray and transparent. (*a*) Definition of several quantities of interest. (*b*) Peak height for three peak types. (*c*) Trough-to-trough durations for three peaks. (*d*) Maximum velocity for three peaks. (*e*) Relationship between peak height and duration. (*f*) Relationship between velocity and duration. (*g*) Relationship between peak height and velocity. (*h*) Mean RMS trough-to-trough curves for co-fluctuation peaks. The inset depicts analogous data but for mean slope, rather than RMS. In this panel, color indicates duration.

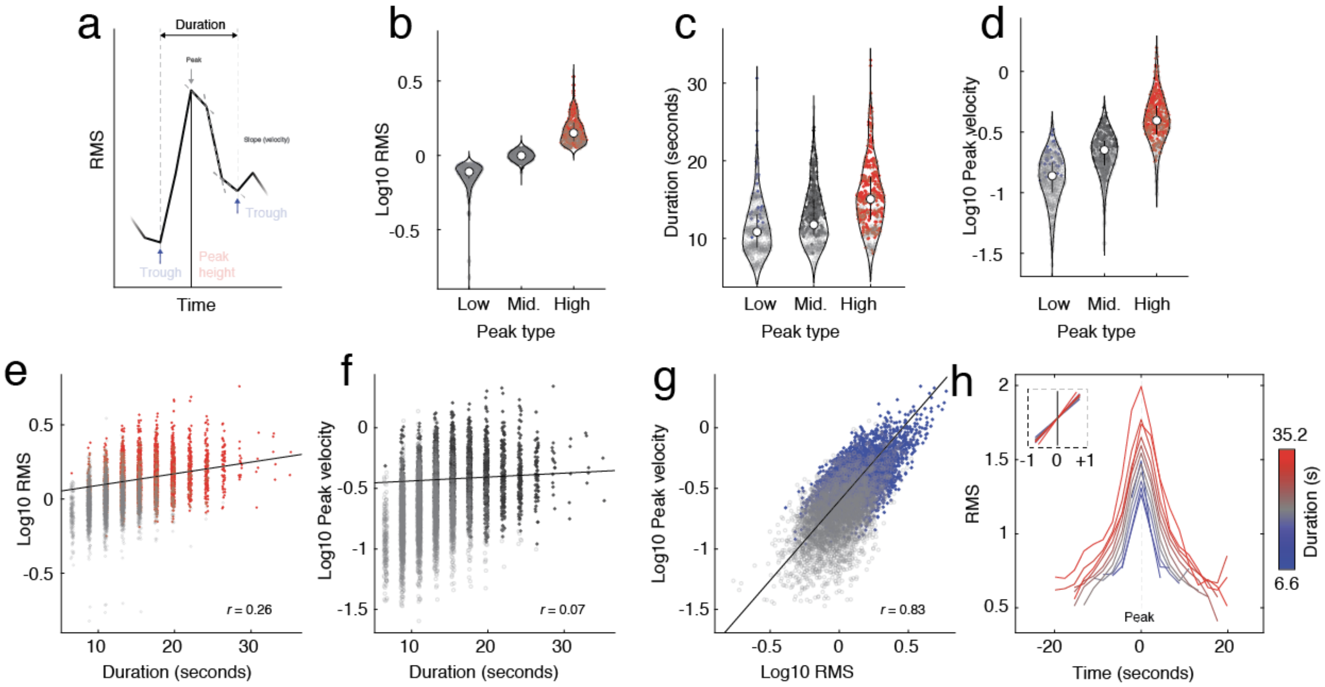

FIG. S3. **Characterizing peak co-fluctuations in Midnight Scan Club data.** In the main text, we characterized peak co-fluctuation using data from the MyConnectome project. Here, we repeated this analysis using data from the Midnight Scan Club after pooling together data from eight subjects (MSC08 and MSC09 were excluded due to data quality issues). For every peak, we calculated its amplitude (RMS) and duration. Using a procedure developed in our previous work, also classified each peak as a high-, middle-, or low-amplitude frame. (*a*) Definition of several quantities of interest. (*b*) Peak height for three peak types. (*c*) Trough-to-trough durations for three peaks. (*d*) Maximum velocity for three peaks. (*e*) Relationship between peak height and duration. (*f*) Relationship between velocity and duration. (*g*) Relationship between peak height and velocity. (*h*) Typical RMS trough-to-trough curves for co-fluctuation peaks. In this panel, color indicates duration.

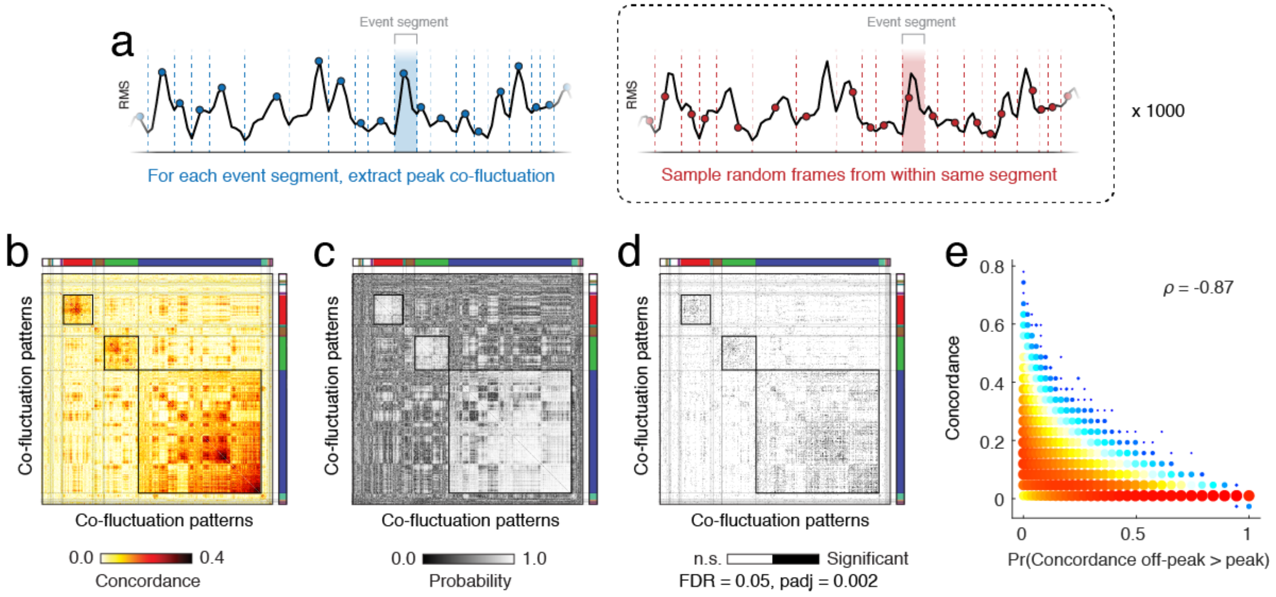

FIG. S4. **Comparing peak and off-peak co-fluctuation patterns.** In the main text, we examine peak co-fluctuation patterns. Here, we compare concordance matrices estimated from peak co-fluctuations against those estimated using patterns taken from within the same event segment but at off-peak frames (see *a* for a schematic of this null model). (*b*) The observed concordance matrix. (*c*) Out of 1000 random samples of off-peak frames, we calculated the fraction of those samples in which the similarity of elements in the null concordance matrix were greater than or equal to those of the observed matrix. Small p-values indicate event segments whose peak-peak concordance was significantly greater than the concordance of random-samples of off-peaks. (*d*) Black cells in this matrix indicate those pairs of segments that survive multiple comparisons corrections (false discovery rate fixed at $q = 0.05$ and the p-value adjusted to $p_{adj} = 0.002$. (*e*) Scatterplot of p-values versus observed concordance values. Note that concordant pairs of co-fluctuations tend to have small p-values.

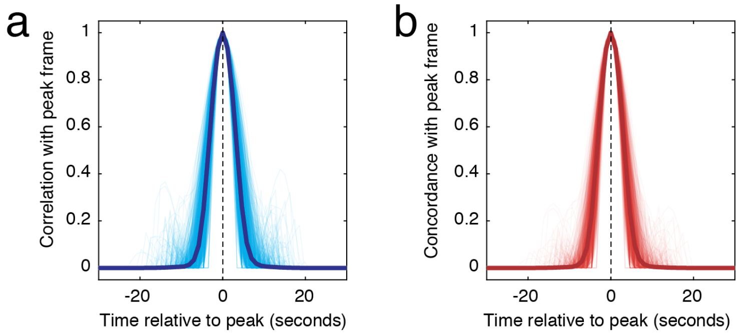

FIG. S5. **Similarity of co-fluctuation patterns to peak.** We analyzed peak co-fluctuation patterns in the main text. Here, we show the similarity of nearby frames in the same segment to those peak patterns. (*a*) Similarity as measured with Pearson's correlation coefficient. (*b*) Similarity as measured by Lin's concordance.

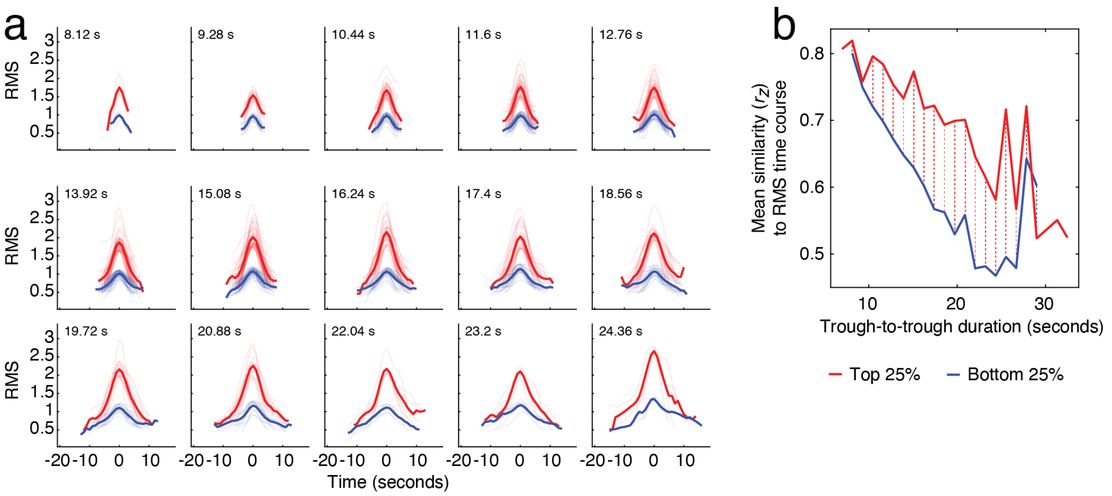

FIG. S6. **Top and bottom RMS quartiles by duration.** In the main text we described a correlation between duration and amplitude of peak co-fluctuations. There was, however, considerable variance around that best linear fit between those variables. (*a*) Here, for each duration (in units of TRs) we show the top (red) and bottom (blue) trough-to-trough curves, ranked by RMS. (*b*) We returned to the edge time series and calculated the correlation of each edge time series with the corresponding trough-to-trough RMS curve. We found that the mean correlation over all edges was stronger for the top 25% than for the bottom 25%, suggesting that even after controlling for duration, there is variability in the "diffusivity" of the trough-to-trough co-fluctuation, with higher amplitude co-fluctuations corresponding to tighter and more cohesive fluctuations than lower-amplitude fluctuations of identical duration.

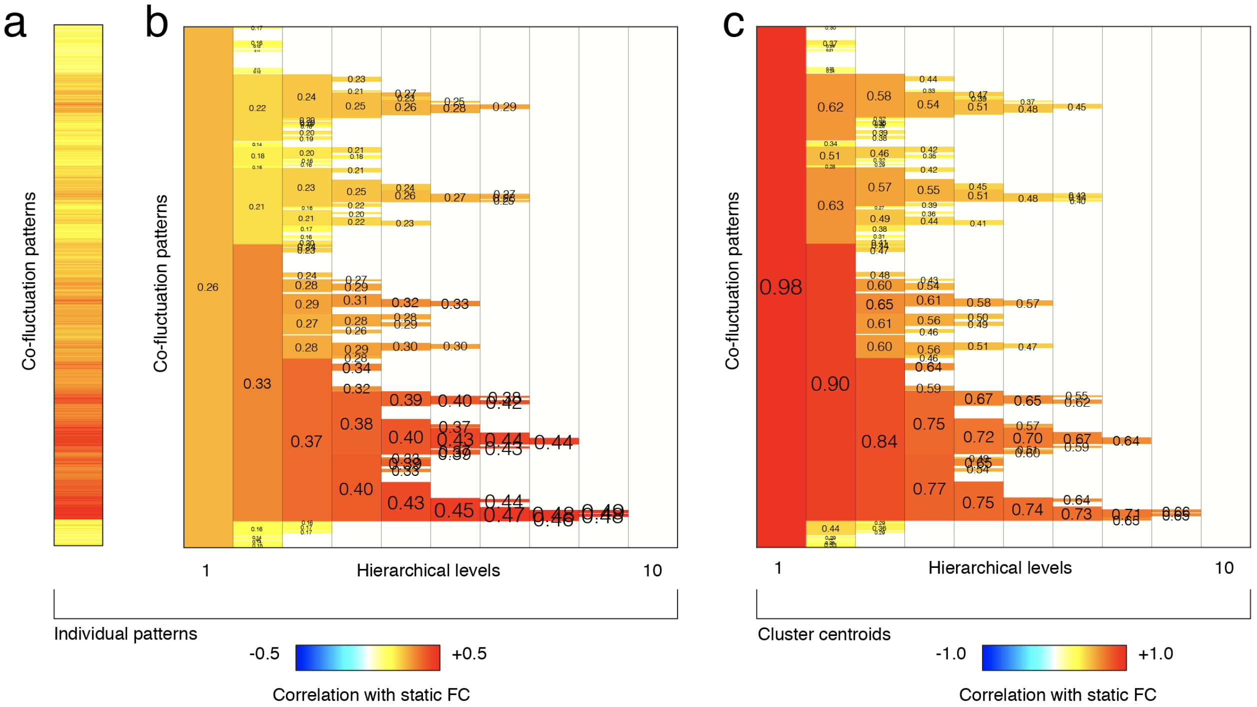

FIG. S7. **Correlation of cluster centroids and individual patterns with static FC.** In Fig. 2j we showed that larger clusters and their centroids were more strongly correlated with FC. Here, we repeat this analysis showing (*a*) the correlation of individual co-fluctuation patterns with FC, (*b*) the mean across those pattern level correlations averaged by cluster, and (*c*) the correlation of cluster centroids with FC.

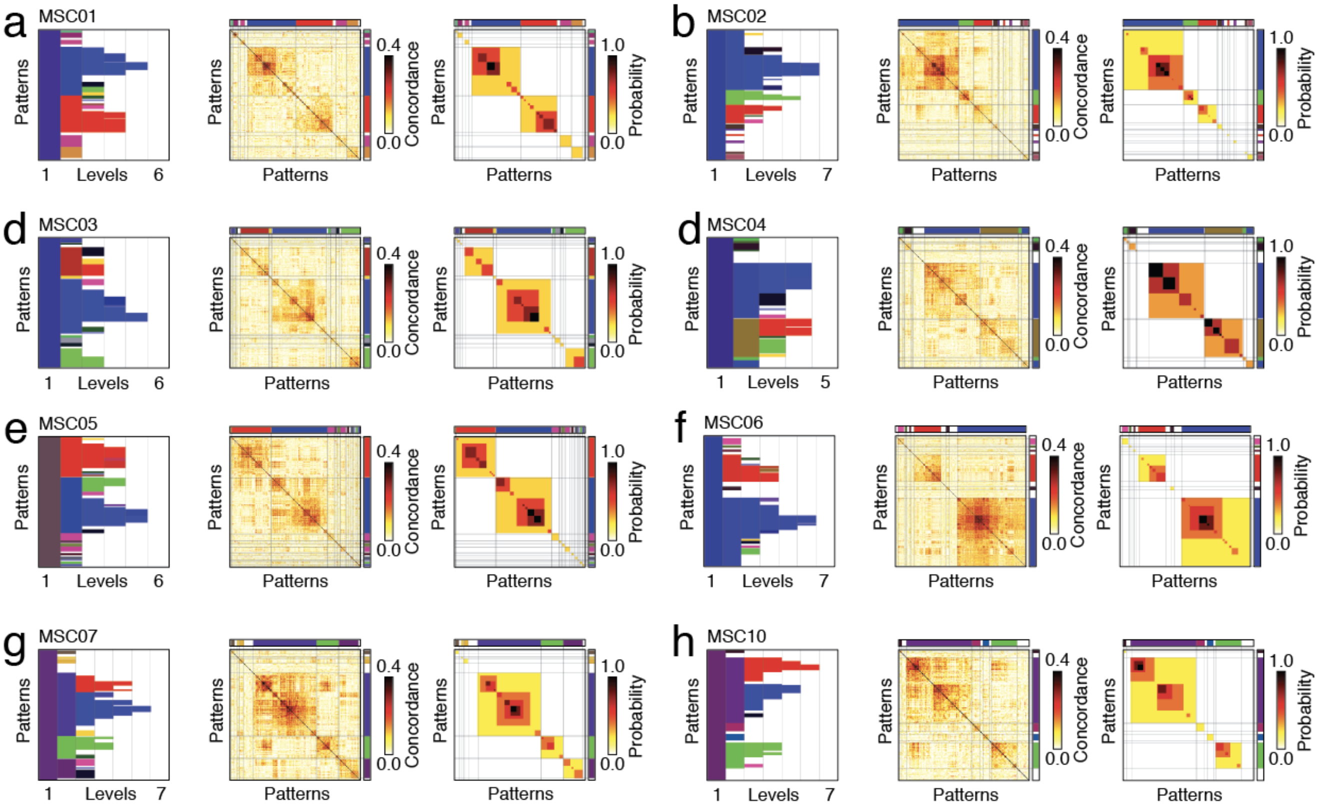

FIG. S8. **Hierarchical clusters, concondance, and co-assignment matrices** Here, we show results of the hierarchical clustering algorithm applied to data from the Midnight Scan Club. Panels *a*-*h* show data from MSC01-MSC07 and MSC10. Each panel includes three sub-panels. From left to right: hierarchical cluster labels for each co-fluctuation pattern; concordance matrix ordered by clusters; co-assignment matrix ordered by clusters.

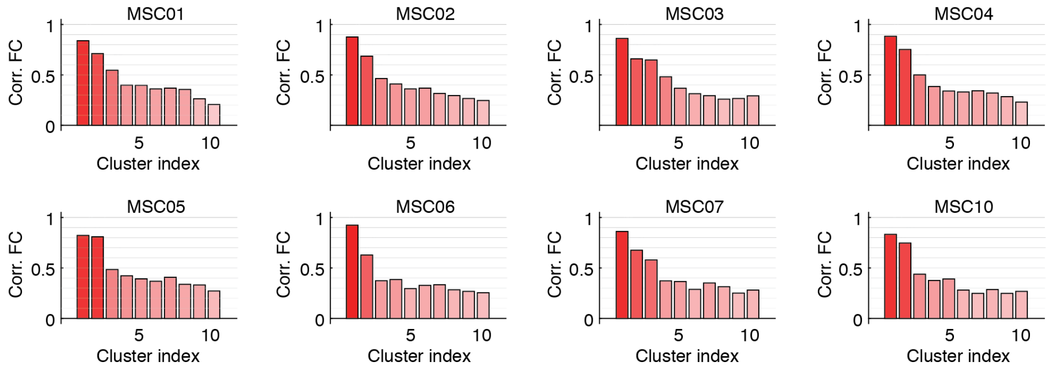

FIG. S9. **Correlation of cluster centroids with static FC.** In Fig. 2j we showed that larger clusters were more strongly correlated with FC. Here, we repeat this analysis using data from the Midnight Scan Club. Each panel correspond to a different subject.

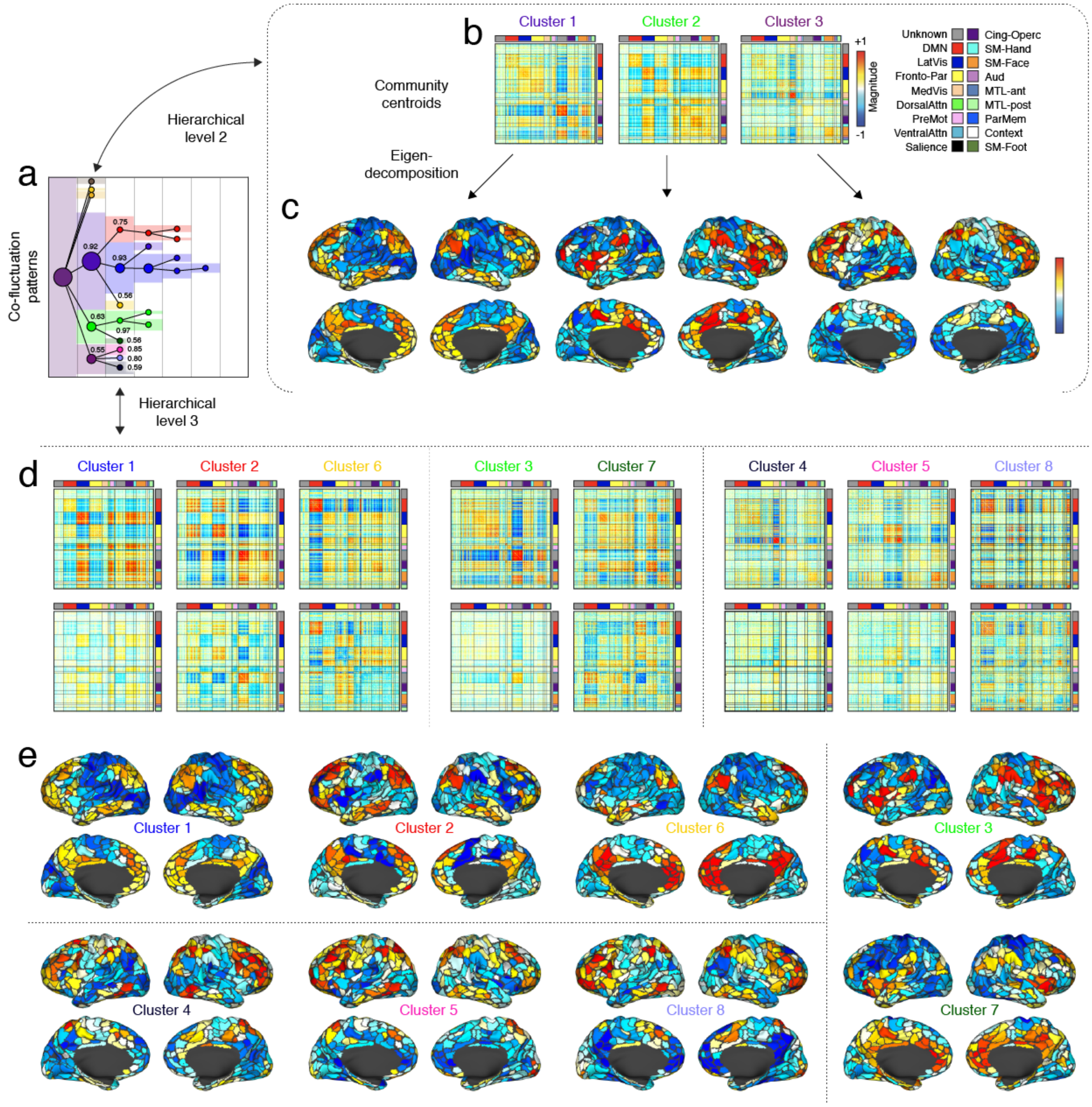

FIG. S10. **Sub-divisions of clusters in MSC data: An example using MSC07.** In the main text we demonstrated that co-fluctuation patterns could be organized hierarchically. In the supplement, we applied the hierarchical clustering algorithm to all MSC participants. For illustrative purposes, we show here the results for subject MSC07. (*a*) Hierarchical cluster assignment with dendrogram overlaid. (*b*) We show the three largest cluster centroids at hierarchical level 2. (*c*) The leading eigenvector for each centroid. (*d*) We show decompositions of clusters 2, 1, and 3 into smaller and more distinct sub-clusters. (*e*) Again, we show the leading eigenvectors for each sub-cluster.

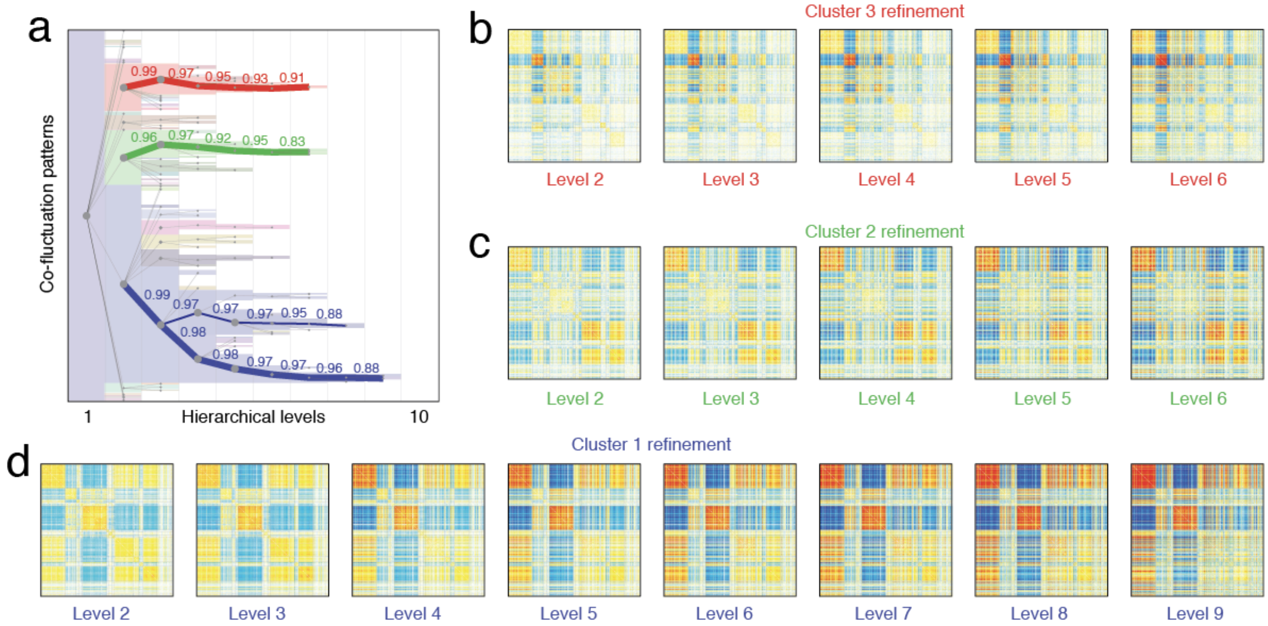

FIG. S11. **Persistence and refinement of coarse clusters across hierarchical levels.** In Fig. 3 and Fig. 4 we showed coarse clusters and their hierarchical divisions. We note that the coarse clusters, although they get sub-divided, are also refined across hierarchical levels. That is, strong co-fluctuations get stronger (positive and negative) but the overall pattern persists. Here, we highlight the persistence of the three large clusters identified in Fig. 3. The correlation values shown in *a* correspond to the correlation of each child centroid with its immediate parent. Note that an alternative possibility was that, as clusters sub-divide, the children partitions decompose their parents so that the correspondence is not as strong.

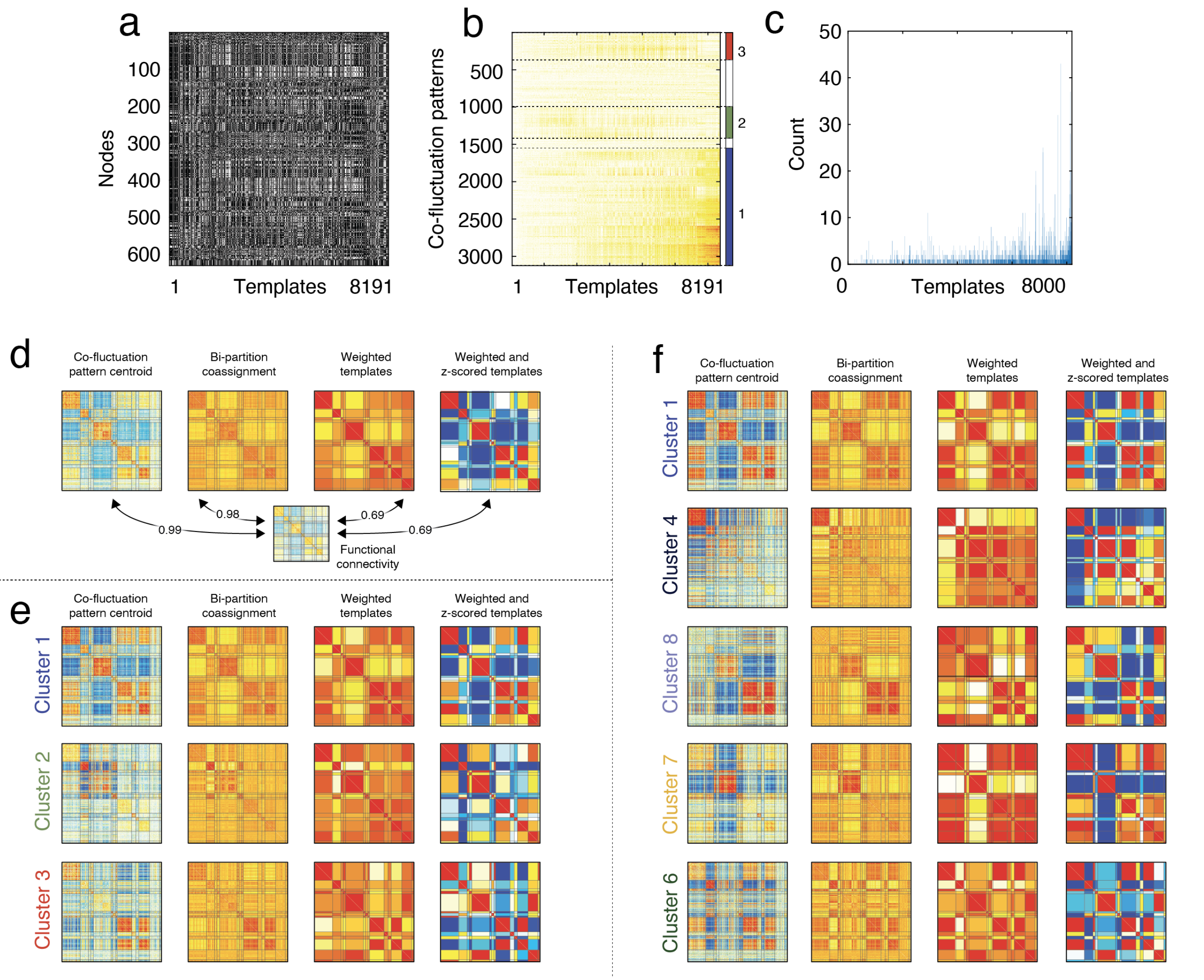

FIG. S12. **Comparison with templates and bipartitions.** (*a*) System templates. Given 14 systems, there are exactly 8191 unique templates–i.e. ways of partitioning systems into two disjoint sets. (*b*) Normalized mutual information of each co-fluctuation pattern with each template. Patterns are ordered by community. Note: Here we include all co-fluctuation patterns, including those with low prominence and without exclusion based on proximity to another peak. (*c*) For each pattern, we identified the index corresponding to the maximum NMI. Here, we show the histogram. (*d*) We then compared different reconstructions of FC to the observed FC matrix. These included the mean co-fluctuation pattern (averaged over all patterns), the co-assignment matrix of observed bipartition, the co-assignment matrix of the best-matched system-templates, and the z-scored version of the best-matched templates. We repeated this analysis for the largest clusters detected at hierarchical level 2 (*e*) and for subdivisions of the largest cluster at that level (*f*). In all cases, we find evidence that templates capture the specificity of divisions among co-fluctuations originally identified using the hierarchical clustering algorithm.

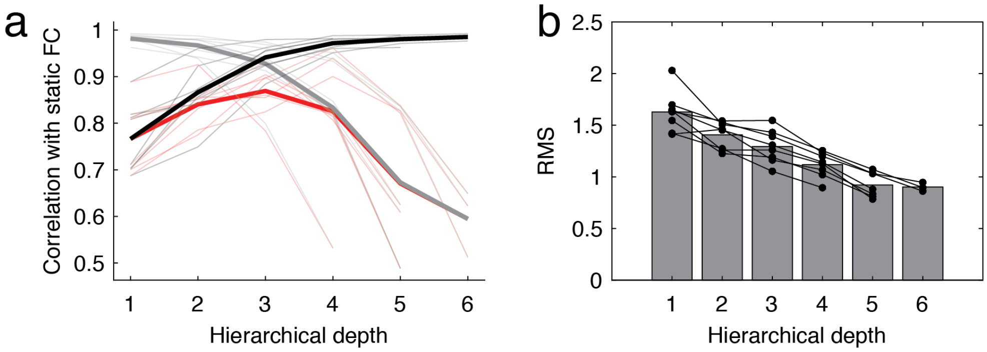

FIG. S13. **Linking different hierarchical levels to FC using MSC data.** In the main text we show that the correspondence with FC peaks at an intermediate hierarchical level. Here, we recapitulate that analysis using data from the individual subjects in the MSC dataset. (*a*) Correlation with FC at different hierarchical levels. Thick lines indicate averages across subjects while thin lines indicate data from individual subjects. (*b*) Mean RMS of co-fluctuation patterns at different hierarchical levels.

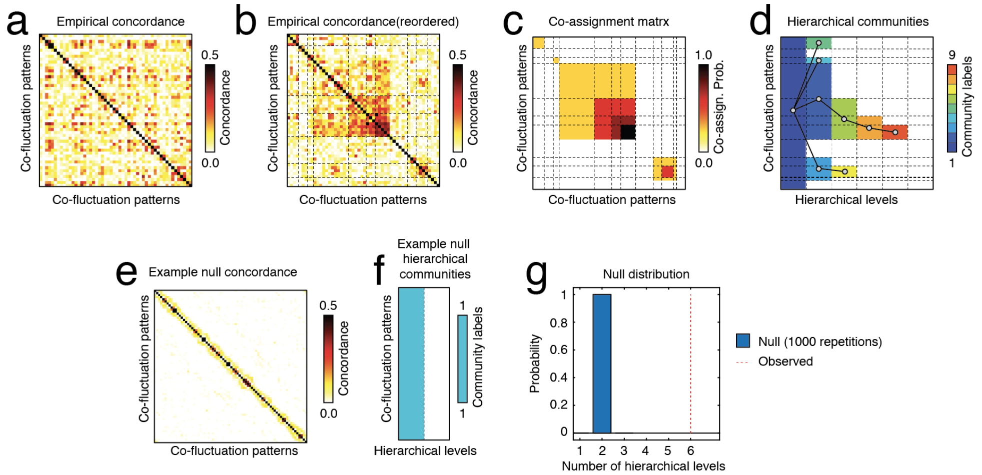

FIG. S14. **Application of hierarchical clustering algorithm to random peaks.** In the main text, we clustered peak co-fluctuation patterns using a bespoke hierarchical modularity maximization algorithm. Here, as an example we cluster the observed peak co-fluctuations from a single scan and compare the results with a null model in which parcel time series were circularly shifted by a random offset prior to computing edge time series (1000 repetitions). (*a*) Concordance matrix between all pairs of observed co-fluctuation patterns. (*b*) Same matrix reordered based on hierarchical communities. (*c*) Co-assignment matrix of co-fluctuation patterns across all hierarchical levels. (*d*) Dendrogram showing hierarchy and divisions of communities. (*e*) An example concordance matrix estimated from a single run of the null model. (*f*) Hierarchical communities. Note that there are only two levels, both of which are trivial. The first level corresponds to a community that contains all nodes; the second level contains no statistically significant communities, i.e. none of the communities in the first level passed a test for statistical significance. We repeated the null model 1000 times and calculated the number of hierarchical levels detected in each run. In all cases, the number of levels was two (2). (*g*) Null distribution concentrated on a single value (blue) compared to the observed number of hierarchical levels (red dashed line).
